# Precision oncology in gynecologic cancers: molecular taxonomy, biomarker-guided therapeutics, and the challenge of therapeutic resistance

**DOI:** 10.3389/fonc.2026.1785469

**Published:** 2026-04-07

**Authors:** Shanza Waseem, Jun Zhan, Xue Xiao, Peng Bai

**Affiliations:** 1 Department of Gynecology and Obstetrics, West China Second University Hospital, Sichuan University, Chengdu, China; 2 Key Laboratory of Birth Defects and Related Diseases of Women and Children (Sichuan University), Ministry of Education, West China Second Hospital, Sichuan University, Chengdu, China; 3 Tianfu Jincheng Laboratory, Chengdu, China; 4 Laboratory of Stem Cell and Embryo Development, West China Second Hospital, Sichuan University, Chengdu, China; 5 Department of Forensic Genetics, West China School of Basic Medical Sciences & Forensic Medicine, Sichuan University, Chengdu, China

**Keywords:** antibody-drug conjugates, gynecologic cancers, immunotherapy, inhibitors, liquid biopsy, molecular classification, PARP

## Abstract

**Introduction:**

The clinical management of gynecologic malignancies endometrial (EC), ovarian (OC), and cervical (CC) carcinomas has been historically guided by histomorphology and staging. This paradigm fails to capture profound molecular heterogeneity, resulting in suboptimal outcomes. Precision oncology, through molecular taxonomy and biomarker-guided therapy, aims to address this gap.

**Methods:**

This review synthesizes the current landscape of precision oncology in gynecologic cancers by analyzing established molecular classifications, the evidence for biomarker-guided therapies, mechanisms of therapeutic resistance, and emerging diagnostic and trial paradigms. The analysis is based on a critical evaluation of key literature and clinical trial data.

**Results:**

Molecular reclassification, exemplified by The Cancer Genome Atlas (TCGA) for EC and OC, has identified prognostically and therapeutically relevant subtypes. This has enabled successful clinical translation of targeted agents: PARP inhibitors for homologous recombination deficient (HRD) ovarian cancer, immune checkpoint inhibitors for mismatch repair deficient/microsatellite instability-high (dMMR/MSI-H) endometrial and cervical cancers, and antibody-drug conjugates (ADCs) like mirvetuximab soravtansine and tisotumab vedotin. However, acquired resistance to these therapies, driven by tumor evolution and heterogeneity, remains a pivotal barrier.

**Conclusion:**

The convergence of deep molecular phenotyping with targeted therapy is a cornerstone of modern care. Future directions require overcoming resistance through novel diagnostics like liquid biopsy, next-generation therapeutics, and innovative adaptive clinical trials. Equitable access to these advances is imperative to prevent widening health disparities. The field is evolving towards a model of continuous molecular monitoring and adaptive therapy to improve outcomes.

## 1 Introduction

Gynecologic cancers represent a substantial global health burden, with 1,473,427 new cases and 680,372 deaths recorded in 2022, demonstrating significant geographic disparities (1). The global incidence rate reached 30.3 per 100,000 women with mortality of 13.2 per 100,000, showing marked regional variations where Eastern Africa exhibited incidence rates exceeding 50 per 100,000 compared to Northern Africa’s 17.1 per 100,000 (1). Country-specific disparities were striking, with Eswatini recording the highest incidence and mortality rates at 105.4 and 71.1 per 100,000 respectively, while Yemen had the lowest at 5.8 and 4.4 per 100,000 (1).

Temporal trends reveal a concerning pattern of increasing burden in low- and middle-income countries (LMICs). Between 2000 and 2022, the global age-standardized incidence rate of gynecologic cancers increased by 8.7%, with the most pronounced rises observed in Sub-Saharan Africa (+21.3%) and South-Central Asia (+17.8%) (1, 2). Conversely, high-income countries have experienced stabilization or modest declines in incidence, largely attributable to HPV vaccination and screening programs (3, 4). Mortality trends similarly diverged: while high-income countries saw a 12.4% reduction in age-standardized mortality over this period, LMICs experienced a 9.6% increase, widening the survival gap (5, 6). These disparities underscore the urgent need for equitable access to prevention, early detection, and precision oncology interventions globally (7, 8).

Projections to 2050 indicate continued divergence, with LMICs expected to bear 85% of the global gynecologic cancer burden compared to 68% in 2022, driven by demographic transitions and limited healthcare infrastructure (1, 9). Cervical cancer, which is preventable through vaccination and screening, accounts for the largest proportion of this disparity, with age-standardized incidence rates in Eastern Africa (56.2 per 100,000) more than 10-fold higher than in Western Asia (5.1 per 100,000) (1, 10).

Traditional therapies comprising cytoreductive surgery, platinum-based chemotherapy, and radiotherapy have demonstrated limited efficacy, particularly in ovarian cancer where chemoresistance represents a major clinical challenge (11). Advanced epithelial ovarian cancer patients who develop chemoresistance face particularly grim prognoses, with expected survival times of less than one year (12). Despite platinum-based chemotherapy remaining standard for over three decades, survival remains uniformly poor for patients with chemoresistant recurrent disease (13).

Precision oncology addresses this by using molecular characterization to identify targetable driver alterations, enabling therapy matching based on genetic events rather than histology (14, 15). This approach leverages high-throughput profiling to discriminate oncogenic “drivers” and facilitate personalized treatment recommendations (15, 16). The framework integrates comprehensive bioinformatics workflows to predict therapeutic response and delineate prognostic subgroups (16, 17).

Large-scale molecular cartography projects, most notably TCGA, have propelled this paradigm shift. TCGA has systematically characterized genomic, epigenomic, and proteomic data from over 10,000 samples across 33 cancer types, providing unprecedented insights (18). These efforts have revealed astonishing diversity within cancer types, dissolving monolithic disease entities into molecularly distinct subtypes with disparate clinical behaviors (18, 19).

In gynecologic oncology, endometrial carcinoma (EC) exemplifies successful clinical translation. The classification of EC into four molecular subgroups POLE-ultramutated, microsatellite instability-high (MSI-H), copy-number high (p53-abnormal), and copy-number low (NSMP) has been formally integrated into WHO and ESGO guidelines (20). This molecular classification has demonstrated significant clinical impact, with studies showing distribution of 7.6% POLE, 32.2% MMRd, 20.9% p53abn, and 39.3% NSMP subtypes, leading to a 32.7% risk group change compared with previous systems (20). Molecular classes are statistically correlated with disease-free survival and guide adjuvant therapy selection (21).

Targeted agents exemplify this precision approach. PARP inhibitors like olaparib, approved for BRCA-mutated ovarian cancer, demonstrate durable antitumor activity in platinum-sensitive disease (22). Clinical trials show epithelial ovarian cancer patients with BRCA mutations exhibit favorable responses to olaparib compared to those without mutations (23).

Nevertheless, implementation faces formidable challenges. Tumor heterogeneity and complex tumor microenvironments foster adaptive therapeutic resistance (24). The voluminous, heterogeneous data from omics, electronic health records, and medical imaging complicate precision approaches (25). Clinical utility of biomarkers like HRD scores requires standardized assay validation, as tests vary significantly in genomic features measured and threshold determination methods (26). HRD test results and PARPi responses can be discordant due to reversion mutations or resistance mechanisms independent of homologous recombination (26). Socioeconomic and infrastructural barriers to comprehensive genomic testing threaten to exacerbate healthcare disparities, with improving equity representing a priority value of 22.6% among policy experts (27).

This review provides a comprehensive critical analysis of precision oncology in gynecologic cancers, detailing: (1) established molecular taxonomies and clinical implications; (2) mechanistic basis and evidence for biomarker-guided therapeutics; (3) emerging resistance mechanisms; and (4) novel diagnostics and adaptive trial designs poised to shape personalized care.

## 2 Materials and methods

### 2.1 Search strategy and study selection

This narrative review was conducted through systematic searches of the peer-reviewed literature. We searched PubMed/MEDLINE, Embase, and the Cochrane Library for articles published between January 2010 and February 2026. The search strategy combined the following keywords and MeSH terms: “precision oncology,” “gynecologic cancer,” “endometrial cancer,” “ovarian cancer,” “cervical cancer,” “molecular classification,” “biomarker,” “PARP inhibitor,” “immunotherapy,” “antibody-drug conjugate,” “therapeutic resistance,” “liquid biopsy,” and “clinical trial design”.

### 2.2 Inclusion criteria

Studies were included if they met the following criteria: (1) pivotal phase II or III clinical trials establishing efficacy of biomarker-guided therapies; (2) landmark genomic studies defining molecular taxonomies (including TCGA and subsequent validation studies); (3) translational research elucidating mechanisms of therapeutic resistance; (4) high-quality systematic reviews and meta-analyses; and (5) articles published in English.

### 2.3 Priority and selection process

Priority was assigned to: (a) studies with the highest level of evidence (randomized controlled trials, prospective validation studies); (b) research that has directly influenced clinical guidelines (National Comprehensive Cancer Network [NCCN], European Society of Gynaecological Oncology [ESGO], American Society of Clinical Oncology [ASCO]); and (c) publications from the past 5 years (2021–2026) to ensure currency. Seminal older papers were included where historically important. Reference lists of key articles were manually screened to identify additional relevant studies.

### 2.4 Ethics approval

As this was a narrative review of published literature, no ethics approval was required.

## 3 Molecular taxonomy of gynecologic cancers: from genomic landscapes to clinical classifiers

Histopathological diagnosis of gynecologic cancers provides limited insight into biological aggressiveness or drug sensitivity, necessitating more sophisticated stratification approaches. The integration of molecular data has led to refined classification systems that better predict disease course and therapeutic vulnerability. Endometrial cancer exemplifies this evolution, with large-scale genomic studies revealing at least four distinct molecular subtypes carrying significant prognostic and predictive information (28). Integrated approaches combining next-generation sequencing and immunohistochemistry enable algorithmic molecular classification of newly diagnosed endometrial cancer, proving prognostic in both early- and advanced-stage disease (29).

This evolution represents a paradigm shift from traditional histopathology toward precision medicine that more accurately guides therapeutic decisions. For instance, in endometrial carcinoma, POLE mutations signal favorable prognoses despite aggressive histological appearance, while copy-number high (CNH) tumors require more intensive therapy (30). This approach enables biomarker-driven treatment, where MMR-deficient endometrial tumors respond to pembrolizumab, while CNH tumors benefit from lenvatinib-pembrolizumab combinations (30). Thus, molecular signatures are shifting oncological practice from morphology-based to biology-guided care, where genetic profiles increasingly direct therapy over traditional pathological classifications.

### 3.1 Endometrial carcinoma: the TCGA legacy and ProMisE implementation

**Figure 1:**
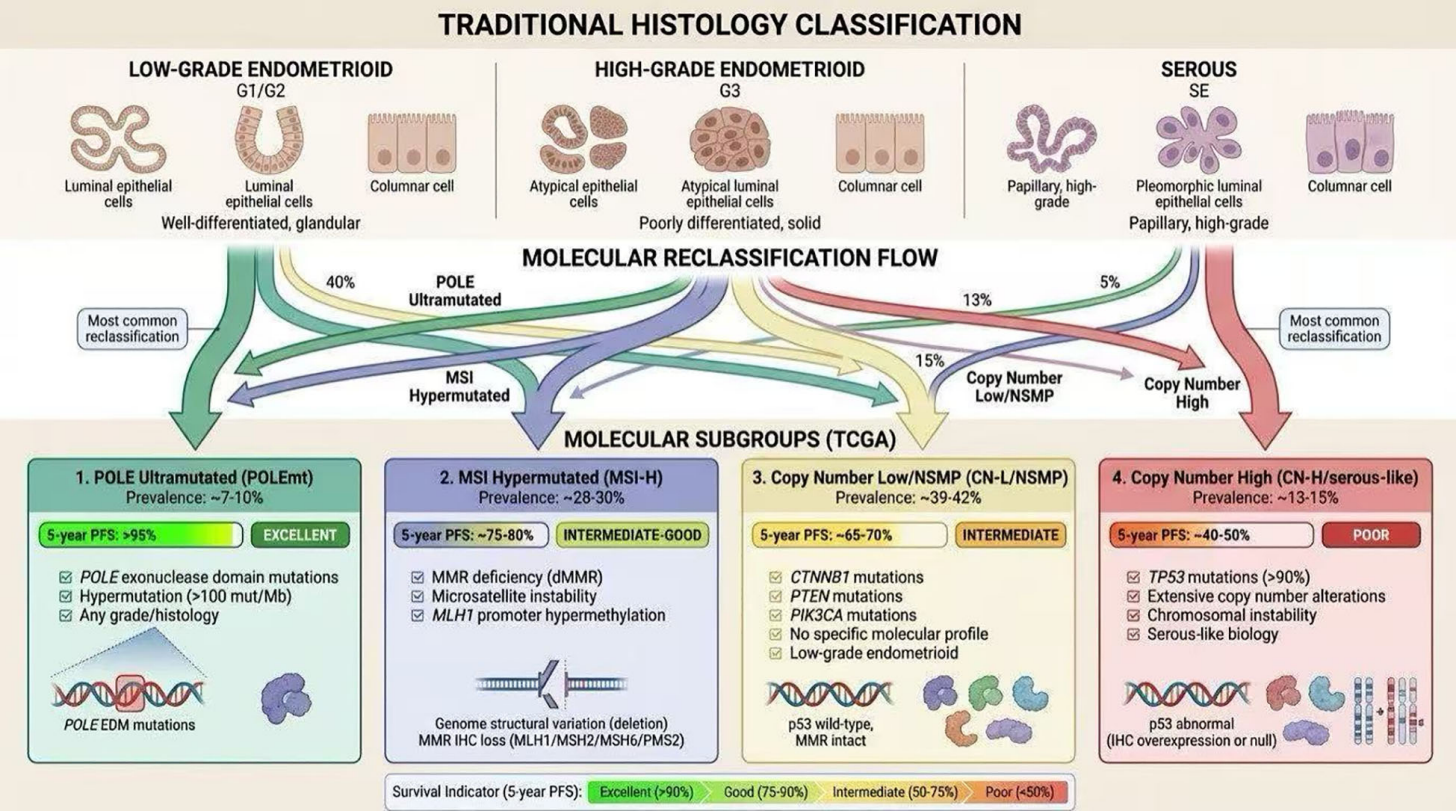
Molecular classification of endometrial carcinoma based on The Cancer Genome Atlas (TCGA) and associated clinical outcomes. The figure illustrates the paradigm shift from histology-based to molecular taxonomy in endometrial cancer. Top panel: Traditional histological classification. Middle panel: Molecular reclassification flow showing redistribution of histologically similar tumors into four TCGA subgroups. Bottom panel: The four molecular subgroups with defining genomic features, prevalence, and 5-year progression-free survival (PFS): (1) POLE-ultramutated (7-10%): POLE exonuclease domain mutations, hypermutation (>100 mutations/Mb), excellent prognosis (>95% 5-year PFS). (2) MSI-H (28-30%): MMR deficiency, intermediate-good prognosis (75-80% 5-year PFS). (3) Copy-number low/NSMP (39-42%): CTNNB1/PTEN/PIK3CA mutations, p53 wild-type, intermediate prognosis (65-70% 5-year PFS). (4) Copy-number high/serous-like (13-15%): TP53 mutations (>90%), chromosomal instability, poor prognosis (40-50% 5-year PFS). The integrated classification provides superior prognostic stratification and guides precision therapy (e.g., immunotherapy for MSI-H/dMMR tumors). EDM, exonuclease domain mutations; IHC, immunohistochemistry; MMR, mismatch repair; NSMP, no specific molecular profile.

The TCGA analysis of 373 endometrial carcinomas identified four molecular subgroups with significantly different clinical outcomes (**Figure 1**) (31). The *POLE*-ultramutated subgroup, characterized by pathogenic exonuclease domain mutations and ultra-high mutation burden, demonstrates an exceptionally favorable prognosis despite often high-grade histology (31). The MSI-H subgroup represents hypermutated tumors with intermediate prognosis. The Copy-Number High/p53-abnormal subgroup, defined by extensive copy-number alterations and frequent TP53 mutations, carries the poorest prognosis, with statistically significant associations between p53 expression and TCGA classification (χ²= 11.585, p = 0.005) (31). The Copy-Number Low subgroup, typically comprising low-grade, estrogen receptor-positive tumors, shows favorable outcomes, with significantly higher survival in ER-positive patients (p < 0.001) (31).

The ProMisE (Proactive Molecular Risk Classifier for Endometrial Cancer) algorithm provides a clinically feasible surrogate for TCGA classification, using sequential immunohistochemistry for p53 and mismatch repair proteins followed by *POLE* sequencing (32). Research demonstrates that molecular testing changes risk category in 89/896 (10%) of endometrial carcinomas (31, 32). The algorithm enables selective testing strategies, with *POLE* testing potentially restricted to 55% of biopsies and 38% of all carcinomas in population-based cohorts (32). This approach fundamentally challenges traditional risk stratification, as patients with *POLE* mutations show superior survival regardless of grade, while p53-abnormal tumors have poor prognosis even with low-grade morphology evident in 15 of 166 (9%) such cases (31, 32).

However, clinical implementation faces significant practical barriers. Survey data reveals a critical disconnect: while over 90% of clinicians deem markers like *POLE*, MMR, and p53 status important, only 24.2% report that *POLE* testing is easy to obtain, highlighting substantial access disparities (33). These barriers including cost, workflow integration, and the need for multidisciplinary education are more pronounced in community settings compared to academic centers (33). The urgent need for standardized guidelines and quality assurance programs is underscored by variability in testing platforms and the evolving nature of classification algorithms, which threaten to compromise equitable implementation across diverse clinical settings (33).

### 3.2 Ovarian carcinoma: a dichotomy of type I and II, refined by genomic instability

Ovarian carcinomas are now recognized as a heterogeneous collection of distinct diseases, fundamentally categorized into Type I and Type II tumors. Type I tumors (e.g., low-grade serous, mucinous, endometrioid, clear cell) exhibit indolent clinical behavior, early-stage detection, and rarely harbor *TP53* mutations (34). They can be subdivided into endometriosis-related tumors, low-grade serous carcinomas, and mucinous carcinomas, each with distinct molecular profiles involving mutations in *KRAS, BRAF, CTNNB1, PTEN, PIK3CA, and ARID1A* (34, 35). In contrast, Type II tumors (comprising ~75% of cases, primarily high-grade serous carcinomas) display aggressive biological behavior, late-stage diagnosis, and harbor *TP53* mutations in ~80% of cases (34). Type I tumors develop from benign extraovarian lesions implanting on the ovary, while Type II carcinomas frequently originate from fallopian tube intraepithelial carcinomas, explaining their aggressive dissemination (35).

TCGA analysis of 489 high-grade serous ovarian carcinomas (HGSOCs) revealed a molecular landscape characterized by near-universal *TP53* mutations (>96%) and pervasive genomic instability, yet lacking other recurrent targetable oncogenic drivers (36). A critical functional consequence is HRD, emerging through germline/somatic *BRCA1/2* mutations, alterations in other HR genes (*RAD51C/D, PALB2*), or epigenetic silencing (37). This deficiency impairs double-strand DNA break repair, creating therapeutic vulnerability exploited by PARP inhibitors through synthetic lethality (37).

HRD assessment employs two primary methodologies: (1) direct *BRCA1/2* mutation testing, and (2) genomic scar assays quantifying loss of heterozygosity (LOH), telomeric allelic imbalance, and large-scale state transitions to generate an HRD score (38, 39). Tumors with HRD score ≥42 or *BRCA* mutations are classified HRD-positive. Patients with high genomic scar scores achieve superior median overall survival with PARP inhibitors (50.8 months for *BRCA*-mutant; 42.7 months for BRCA-wild type) versus low-score patients (36.6 months) (39).

Despite the clinical utility of HRD testing, significant limitations and ongoing debates regarding assay standardization warrant critical discussion. Currently, three FDA−approved companion diagnostics are available for PARP inhibitors Myriad myChoice CDx, FoundationOne CDx, and AmoyDx HRD Focus Panel each employing different genomic scar metrics and threshold definitions, and technical platforms (40–44). **Table 1** provides a comprehensive comparison of these assays, highlighting the substantial variability in their methodologies, performance characteristics, and clinical validation.

**Table 1:** Comprehensive comparison of FDA-approved HRD assays for PARP inhibitor selection.

Characteristic	Myriad myChoice CDx	FoundationOne CDx	AmoyDx HRD focus panel
Manufacturer	Myriad Genetics (42)	Foundation Medicine (43)	Amoy Diagnostics (40, 44)
FDA/Regulatory Scope (ovarian HRD use)	FDA-approved CDx for olaparib + bevacizumab in HRD-positive tumors; reference assay in trials (42)	FDA-approved CDx for BRCA1/2 tumor alterations; reports genomic LOH (gLOH) (42, 43)	CE-IVD/regional approvals; validated against Myriad reference (40, 44)
Platform Technology	Custom array CGH + NGS genomic scar assay (GIS) (42)	Hybrid-capture NGS with genome-wide LOH fraction (43)	Targeted NGS with integrated genomic scar algorithm (40, 44)
Genomic Coverage	Genome-wide SNPs to compute LOH, TAI, LST (42)	~300+ genes; computes genome-wide LOH fraction (43)	40+ HRR genes + SNPs for genomic scars (40, 44)
Genomic Scar Metrics	LOH + TAI + LST composite (GIS) (42)	LOH fraction only (43)	LOH + TAI + LST composite (GIS) (40, 44)
Positivity Threshold	GIS ≥42 (40, 42, 45)	LOH ≥16% (commonly used) (43)	GIS ≥42 (validated vs. Myriad) (40, 44)
*BRCA* Status Integration	*BRCA1/2* included in overall HRD call (42)	*BRCA1/2* pathogenic variants HRD-positive regardless of LOH (42)	*BRCA1/2* integrated into HRD call (40, 44)
Sample Requirements	FFPE; ≥20% tumor; ≥50 ng DNA (typical) (46, 47)	FFPE; ≥20% tumor; ≥50 ng DNA (typical) (43)	FFPE; ≥30% tumor; ≥100 ng DNA (reported) (40)
Turnaround Time	~10–14 days (centralized) (44, 48)	~7–10 days (single-platform CGP) (43)	~7–10 days (local lab) (44, 49)
Concordance vs. Myriad (overall)	Reference standard (42)	74–90% across cutoffs; higher in BRCA-mut (49, 50)	84–89% overall; NPV up to 100% (40, 44)
Concordance in *BRCA*-mut	Reference standard (42)	~98% with reference (43, 51)	~97% with Myriad (40)
Concordance in *BRCA*-wt	Reference standard (42)	~68–73% (LOH-only) (43, 52)	~73–89% vs. Myriad (44, 49)
Concordance in intermediate scores (30–41)	N/A (40, 53)	~50–60% (44)	~55–60% (44)
Validation link to trials	PAOLA-1, PRIMA (42)	Retrospective LOH in PAOLA-1, PRIMA (42, 43)	PRIME; multi-center validations vs. Myriad (40, 44, 53)
PFS HR in HRD+ (vs placebo)	0.33 (PAOLA-1); 0.43 (PRIMA) (42)	0.39 (PAOLA-1); 0.40 (PRIMA) (42)	0.38 (PRIME) (40)
PFS HR in HRD− (vs placebo)	0.92 (PAOLA-1); 0.68 (PRIMA) (42)	0.88 (PAOLA-1); 0.65 (PRIMA) (42)	0.85 (PRIME) (40)
HRD+ Prevalence in HGSOC	~48–52% (41, 54)	~42–46% (LOH ≥16%) (43)	~47–51% (40, 44)
Notable Advantages	Most validated; reference in registrations (42)	Single-platform CGP; detects other drivers (43)	Decentralized; faster local TAT; high NPV (44, 49)
Key Limitations	High cost; centralized; not CGP (55, 56)	LOH-only may miss HRD; lower concordance in indeterminate range (26, 57)	Fewer global validations; higher DNA input; limited non-Asian data (40, 44, 58)

As shown in **Table 1**, the concordance between assays is suboptimal, with overall agreement ranging from 74–90% for FoundationOne CDx versus Myriad myChoice CDx (49, 50), and 84–89% for AmoyDx HRD Focus Panel versus Myriad myChoice CDx (40, 44). This discordance is most pronounced in tumors with intermediate genomic instability scores (scores 30–41), where concordance drops to 50–60% (44).

A recent multi-assay comparison reported substantial but incomplete agreement across platforms, with overall percent agreement ranging from ~78% to 89% versus myChoice and notable misclassification at intermediate genomic instability scores, underscoring real-world discordance in HRD calls (40, 50). In practical terms, this variability can reclassify a meaningful minority of patients; studies show alternative assays may label roughly 10–15% of tumors differently from the reference test, potentially altering access to PARP inhibitor therapy despite similar clinical enrichment for benefit in HRD-positive groups (50, 53).

Importantly, the predictive validity of these assays varies by clinical context. In *BRCA*-mutant tumors, concordance is excellent (>97–98%), and all assays reliably predict PARP inhibitor benefit with PFS hazard ratios of 0.33–0.43 in pivotal trials (40, 42). However, in the ‘HRD non-BRCA’ population tumors with genomic instability but no BRCA mutation the predictive value is attenuated (PFS HR 0.40–0.50), and assay discordance can significantly impact treatment decisions (40, 42). The table also highlights important practical considerations, including sample requirements, turnaround times, cost, and geographic availability, which influence real-world implementation (55–58).

Emerging data also highlight the dynamic nature of HRD status. Serial analysis of circulating tumor DNA shows HRD can shift over time via *BRCA1/2* reversion mutations, epigenetic reversion of *BRCA1* methylation, or selection of HR−proficient subclones, with reversions sometimes detectable before clinical progression (59). This temporal variability challenges single−timepoint testing and supports longitudinal monitoring (59).

Approximately 20–30% of tumors with high HRD scores exhibit primary resistance to PARP inhibitors, underscoring that genomic scarring reflects historical, rather than current, HR function (60–62). Functional HRD assays such as RAD51 foci formation or related immunohistochemical readouts aim to capture real-time HR status and have shown prognostic and predictive value, though standardization challenges and variable failure rates remain significant hurdles (63, 64).

The clinical utility of HRD testing also varies by ovarian cancer histotype. HRD is common in high-grade serous ovarian carcinoma (approximately 48–52%), but markedly less frequent in clear cell (15–20%), endometrioid (10–15%), and mucinous carcinomas (<5%), supporting integration with detailed pathology review to avoid misclassification (65).

Optimal timing of HRD testing remains debated. Because genomic scar assays reflect historical, rather than current, homologous recombination status, some evidence favors assessing HRD on fresh or recently collected tissue. Functional assays (e.g., RAD51 foci) aim to capture real-time status but face variable failure rates and standardization hurdles, which can impact predictive accuracy when using archival material (26, 62).

Multi-omics subtyping has further dissected HGSOC heterogeneity. Studies have defined microenvironment-based subtypes: immune-desert (CS1), immune/non-stromal (CS2), and immune/stromal (CS3) (66). These subtypes hold direct prognostic and predictive value: the CS2 subtype correlates with the best overall survival and highest predicted response to immunotherapy, whereas the CS3 subtype, despite poor prognosis and low immunotherapy response, shows predicted sensitivity to PARP and VEGFR inhibitors (66). Additional classifications, such as HRR-activated and mesenchymal subtypes, further stratify outcomes, with the mesenchymal subtype (characterized by epithelial-to-mesenchymal transition) linked to significantly worse survival (67). Broader integrative analyses have delineated up to seven reproducible HGSOC subtypes, each with unique multi-omic profiles and activated biological pathways, including cell cycle, immune signaling, and EMT (68).

This evolution from a dualistic histological model to a refined molecular taxonomy enables subtype-specific therapeutic predictions, moving beyond a one-size-fits-all approach. It establishes a critical foundation for precision medicine, where treatment is guided by the underlying molecular drivers and tumor microenvironment of an individual’s HGSOC (66, 68).

As summarized in **Table 2**, key biomarkers guiding targeted therapy in gynecologic cancers include HRD for PARP inhibitors in ovarian cancer (69, 70); dMMR/MSI-H for immune checkpoint inhibitors in endometrial and cervical cancers (71, 72); NTRK fusions for TRK inhibitors across gynecologic cancers (rare incidence) (73); and HER2 overexpression/amplification for HER2-targeted therapies in uterine serous carcinoma (73). Furthermore, the high expression of FRα and Tissue Factor (CD142) are validated predictive biomarkers for the antibody-drug conjugates mirvetuximab soravtansine in platinum-resistant ovarian cancer and tisotumab vedotin in recurrent/metastatic cervical cancer, respectively (73, 74).

**Table 2:** Biomarker definitions and associated targeted therapies in gynecologic cancers.

Biomarker	Molecular definition	Primary detection methods	Relevant cancer types	Associated targeted therapy	Key clinical trial(s)	Reference(s)
HRD	Deficiency in the homologous recombination DNA repair pathway	Germline/somatic *BRCA1/2* sequencing. Genomic Scar Score (LOH, TAI, LST).	Ovarian (HGSOC)	PARP inhibitors (Olaparib, Niraparib, Rucaparib)	SOLO-1, PRIMA, ARIEL3	(69, 70)
dMMR/MSI-H	Defective DNA mismatch repair; high microsatellite instability	IHC for MMR proteins (MLH1, MSH2, MSH6, PMS2). PCR for MSI. NGS.	Endometrial, Cervical	Immune Checkpoint Inhibitors (Pembrolizumab, Dostarlimab)	KEYNOTE-158, GARNET	(71, 72)
NTRK Fusion	Oncogenic gene fusion involving *NTRK1*, *NTRK2*, or *NTRK3*	RNA-based NGS. Pan-TRK IHC (screening).	All (rare incidence)	TRK inhibitors (Larotrectinib, Entrectinib)	NAVIGATE, STARTRK-2	(73)
HER2 Overexpression	*ERBB2* gene amplification or protein overexpression	IHC (3+ or 2+ with ISH confirmation). FISH/NGS for amplification.	Endometrial Serous Carcinoma	HER2-targeted therapy (Trastuzumab, T-DXd)	NCT05256225	(73)
FRα (Folate Receptor Alpha) High Expression	Expression on ≥75% of tumor cells with ≥2+ staining intensity (by specific IHC assay)	IHC (with specific scoring criteria).	Platinum-Resistant Ovarian Cancer	Mirvetuximab soravtansine (ADC)	MIRASOL	(70, 74)
Tissue Factor (TF/CD142)	High expression of tissue factor	IHC.	Recurrent/Metastatic Cervical Cancer	Tisotumab vedotin (ADC)	innovaTV 204	(70, 74)

*HGSOC*, High-Grade Serous Ovarian Carcinoma; *HRD*, Homologous Recombination Deficiency; *LOH*, Loss of Heterozygosity; *TAI*, Telomeric Allelic Imbalance; *LST*, Large-Scale State Transitions; *dMMR*, Deficient Mismatch Repair; *MSI-H*, Microsatellite Instability-High; *IHC*, Immunohistochemistry; *NGS*, Next-Generation Sequencing; *FISH*, Fluorescence In Situ Hybridization; *ADC*, Antibody-Drug Conjugate. *BRCA*, BReast CAncer gene; *BRCA-mut*, BRCA-mutated; *BRCA-wt*, BRCA-wildtype; *CDx*, Companion Diagnostic; *CE-IVD*, Conformité Européenne - In Vitro Diagnostic; *CGH*, Comparative Genomic Hybridization; *CGP*, Comprehensive Genomic Profiling; *DNA*, Deoxyribonucleic Acid; *FDA*, Food and Drug Administration; *FFPE*, Formalin-Fixed Paraffin-Embedded; *GIS*, Genomic Instability Score; *gLOH*, genomic Loss of Heterozygosity; *HGSOC*, High-Grade Serous Ovarian Carcinoma; *HR*, Hazard Ratio; *HRD*, Homologous Recombination Deficiency; *HRR*, Homologous Recombination Repair; *LOH*, Loss of Heterozygosity; *LST*, Large-Scale State Transitions; *N/A*, Not Applicable; *ng*, nanogram; *NGS*, Next-Generation Sequencing; *NPV*, Negative Predictive Value; *PARP*, Poly (ADP-ribose) Polymerase; *PFS*, Progression-Free Survival; *PRIMA*, PRIMA/ENGOT-OV26/GOG-3012 trial; *PRIME*, PRIME/ENGOT-OV26/GOG-3012 trial (Asian population); *SNP*, Single Nucleotide Polymorphism; *TAI*, Telomeric Allelic Imbalance; *TAT*, Turnaround Time.

### 3.3 Cervical carcinoma: beyond HPV integration

While persistent infection with high-risk human papillomavirus (HPV) is the necessary causative agent for most cervical cancers, tumor progression is driven by subsequent host genomic alterations. For instance, *PIK3CA* mutations occur in approximately 60% of cervical squamous cell carcinomas (75). Similarly, in HPV-positive vulvar squamous cell carcinomas a related malignancy *PIK3CA* alterations are present in 31% of tumors, alongside *PTEN* mutations (14%) and *EP300* mutations (14%) (76). These findings underscore that while HPV initiates carcinogenesis, the accumulation of specific host genomic alterations critically determines tumor aggressiveness and potential therapeutic vulnerabilities.

Recent comprehensive molecular characterization has further revealed significant genomic complexity. Whole-exome sequencing of 228 primary cervical cancers identified frequent mutations in epigenetic regulators and the NOTCH pathway, as well as key immune evasion genes (77). The study highlighted HPV integration as a major oncogenic mechanism, observed in all HPV18-related samples and 76% of HPV16-related samples (77). Critical findings included amplifications in the immune checkpoint genes *CD274 (PD-L1)* and *PDCD1LG2 (PD-L2)*, and novel significantly mutated genes such as *SHKBP1, ERBB3, CASP8, HLA-A, and TGFBR2* (77).

Integrative clustering of these data delineated three primary molecular subtypes including keratin-low squamous, keratin-high squamous, and an adenocarcinoma-rich subgroup each with distinct biological features and therapeutic implications (77). These insights are directly informing a paradigm shift in clinical trial design, enabling patient stratification based on molecular features (such as specific driver mutations or immune microenvironment composition) rather than histology alone. This approach holds significant promise for improving therapeutic outcomes by matching targeted agents and immunotherapies to the underlying biology of individual tumors (77, 78).

### 3.4 Emerging molecular classifiers: multi-omic integration and artificial intelligence

The advancement of molecular taxonomy now hinges on integrating genomic, transcriptomic, epigenomic, proteomic, and metabolomic data to build comprehensive molecular portraits, uncovering biological relationships missed by single-omics methods. This multi-omics approach elucidates the functional consequences of genomic changes, with integrated classifiers achieving high accuracy (AUC 0.81-0.87) for difficult tasks like early detection (79). Artificial intelligence (AI), especially deep learning, is essential for the scalable and non-linear synthesis of these diverse data layers into clinical insights using advanced techniques such as graph neural networks and transformers (79). AI-driven analysis has successfully uncovered novel epigenetic subtypes in endometrial cancer linked to prognosis and identified proteomic-level therapeutic targets, such as specific signaling networks and post-translational modifications (79, 80).

AI and machine learning are pivotal for analyzing complex multi-omic datasets to derive new molecular classifications, detecting subtle patterns that may reveal previously unrecognized subtypes (80). A transformative application is computational pathology, where AI analysis of standard H&E slides can predict molecular subtypes, potentially expanding access to precision classification where genomic testing is not feasible (80).

However, deploying AI-based classifiers demands rigorous validation, standardization, and careful attention to ethical challenges like algorithmic bias and transparency, as no AI-derived biomarker currently meets the highest levels of clinical evidence (80). Despite hurdles including data availability and regulatory pathways, these technologies hold significant promise for refining molecular taxonomy and broadening the reach of precision oncology (80, 81).

## 4 Biomarker-guided therapeutic strategies: from synthetic lethality to immune re-engagement

The molecular stratification of gynecologic cancers has enabled the clinical implementation of targeted therapies, marking a paradigm shift from traditional, histology-based approaches to biomarker-directed precision oncology. A pivotal application is immune re-engagement through immune checkpoint inhibitors (ICIs). In cervical cancer, PD-1 blockade combined with platinum-based chemotherapy improves survival in recurrent or metastatic disease (82). Similarly, patients with dMMR/MSI-H endometrial cancer achieve significantly better outcomes with PD-1 blockade (82).

The efficient clinical validation of such biomarker-guided therapies requires strategic trial design, informed by the strength of preliminary evidence and biomarker prevalence (83, 84). Enrichment designs recruiting only biomarker-positive patients are optimal when robust data suggests benefit is confined to a specific subgroup. Conversely, biomarker-stratified designs, which enroll all patients but pre-specify subgroup analysis, are preferred when evidence indicates a greater, but not exclusive, benefit in biomarker-positive patients (85). A full spectrum of methodologies, from retrospective analyses of RCTs to prospective adaptive designs, must be employed to translate biomarkers into validated clinical tools (84).

### 4.1 Exploiting DNA repair deficiencies: PARP inhibitors in ovarian cancer

PARP inhibitors (PARPi) exploit DNA repair deficiencies through synthetic lethality. Approximately 50% of high-grade serous ovarian cancers (HGSOCs) exhibit HRD, making them susceptible to this approach (86). PARPi trap PARP enzymes on damaged DNA, causing replication fork stalling that HR-deficient cells cannot repair (87–89).

Three PARPi olaparib, niraparib, and rucaparib are FDA-approved for ovarian cancer, marking a breakthrough in targeted therapy (88, 89). Their clinical development has evolved from later-line to first-line maintenance therapy. The SOLO-1 trial demonstrated unprecedented efficacy in patients with germline *BRCA* mutations, with a median progression-free survival (PFS) of 56.0 months versus 13.8 months with placebo (hazard ratio [HR] 0.30; 95% confidence interval [CI] 0.23-0.41; *P < 0.001*) (90). The PRIMA trial extended this benefit to the broader HRD-positive population, showing niraparib achieved a median PFS of 21.9 months versus 10.4 months with placebo (HR 0.40; 95% CI 0.27-0.62; *P < 0.001*) (90). These trials established HRD status as the critical predictive biomarker for PARPi benefit.

Combination strategies are reshaping PARP inhibitor (PARPi) therapy in ovarian cancer. In platinum-resistant disease, olaparib plus the ATR inhibitor ceralasertib showed tolerability but limited RECIST responses in the CAPRI platinum−resistant cohort (0% ORR; median progression−free survival 4.2 months), with signals of activity mainly in BRCA1−mutated tumors, tempering expectations for broad homologous recombination–proficient benefit (91). In a separate CAPRI cohort of acquired PARPi−resistant, HR−deficient, platinum−sensitive HGSOC, olaparib ceralasertib achieved a 50% objective response rate, supporting ATR−mediated resensitization after PARPi resistance (92). Preclinical work reinforces this rationale: ATR/CHK1 pathway inhibition can overcome olaparib resistance in BRCA−mutant models and synergize to reduce viability and clonogenic survival, altering DNA damage response protein expression (93, 94).

Anti−angiogenic combinations remain pivotal: the olaparib–bevacizumab strategy is established in HRD−positive advanced ovarian cancer, while cediranib–olaparib demonstrates activity across platinum−sensitive and −resistant settings, with ORR 77% and 23%, respectively, and PFS up to 16.4 months in platinum−sensitive disease, independent of HRR mutation status (95). Mechanistically, combined PARP–ATR inhibition potentiates genome instability and cell death in ATM−deficient contexts, providing a biologic foundation for multi−targeted synthetic lethality approaches (96).

However, resistance remains a challenge. To overcome this, combination strategies are a major research focus, integrating PARPi with antiangiogenics, immunotherapies, or other targeted agents like phosphoinositide 3-kinase inhibitors to enhance efficacy, particularly in homologous recombination proficient tumors (97, 98). Future patient selection may be refined through comprehensive genomic evaluation and standardized HRD assays (97).

### 4.2 Harnessing the immune system: checkpoint inhibition in hypermutated cancers

Tumors with dMMR/MSI-H exhibit a hypermutated phenotype, generating a high burden of immunogenic neoantigens. This often triggers compensatory upregulation of immune checkpoint proteins like PD-1/PD-L1, which suppress the host T-cell response. Immune checkpoint inhibitors (ICIs) block this interaction, reactivating anti-tumor immunity (99). The immunogenicity of this subset is evidenced by PD-L1 positivity in 18.6% of dMMR cancers (vs. 4.1% in MMR-proficient tumors) and significantly higher intratumoral CD8+ T-cell densities, which correlate with response to PD-1 blockade (99, 100).

Pembrolizumab received tumor-agnostic FDA approval for advanced MSI-H/dMMR solid tumors based on the KEYNOTE-158 trial, where updated results with longer follow-up demonstrated an objective response rate (ORR) of 50% (95% CI 40-61%) in patients with MSI-H/dMMR endometrial cancer (EC), compared to only 7% (95% CI 3-14%) in the non–MSI-H/non–dMMR cohort (101). Similarly, dostarlimab achieved an ORR of 45.5% in dMMR/MSI-H EC versus 15.4% in mismatch repair proficient/microsatellite stable (MMRp/MSS) EC in the GARNET trial (102). These results firmly establish MMR/MSI status as a critical predictive biomarker for ICI response.

In cervical cancer, PD-L1 expression (CPS) is a useful but imperfect predictor: benefit from pembrolizumab plus chemotherapy is seen across the overall population, yet appears greater in PD-L1–positive disease, driving regulatory restrictions in many regions (103). In KEYNOTE-826, first-line pembrolizumab plus chemotherapy (± bevacizumab) improved PFS (HR 0.65; 95% CI 0.53–0.79) and OS (HR 0.64; 95% CI 0.50–0.81) in the ITT cohort, with sequential testing confirming benefit in CPS ≥1 and CPS ≥10 subgroups, supporting PD-L1 enrichment but not absolute exclusivity of benefit (103). Health technology assessments recommend use in adults with CPS ≥1, reflecting the stronger signal in PD-L1–positive tumors (104). Approximately 80–85% of advanced cervical cancers are PD-L1 positive (CPS ≥1), aligning with the immune-inflamed milieu of persistent HPV infection; this high prevalence means restricting therapy to PD-L1–positive tumors excludes relatively few patients, yet PD-L1 negativity does not eliminate the chance of benefit (105). In KEYNOTE-158, pembrolizumab monotherapy produced an ORR of 12.2%, with responses enriched in PD-L1–positive tumors (14.6%) versus rare responses in PD-L1–negative disease, highlighting PD-L1 as an enrichment, not absolute, biomarker (105). Meta-analytic data corroborate frequent PD-L1 expression and its association with outcomes (106).

**Table 3** summarizes pivotal immunotherapy trials in cervical cancer stratified by PD-L1 expression. In the first-line setting, KEYNOTE-826 demonstrated that benefit magnitude increases with higher PD-L1 expression: PFS HR improved from 0.72 (95% CI 0.52–1.00) in CPS <1 to 0.62 (95% CI 0.50–0.77) in CPS ≥1, and further to 0.58 (95% CI 0.44–0.77) in CPS ≥10 (47, 103). BEATcc reported PFS HR 0.71 (95% CI 0.49–1.03) for CPS <1 versus 0.60 (95% CI 0.45–0.80) for CPS ≥1 with atezolizumab-based therapy (105). In the previously treated setting, EMPOWER-Cervical 1 demonstrated cemiplimab improved survival regardless of PD-L1 status, though benefit was more pronounced in PD-L1–positive tumors (ORR 18% vs. 11%; PFS HR 0.73 vs. 0.92) (104). The phase II KEYNOTE-158 established pembrolizumab activity in recurrent cervical cancer, with responses confined almost exclusively to PD-L1–positive tumors (14.6% vs. 0%), though the small number of PD-L1–negative patients (n=16) limits definitive conclusions (105, 107). Smaller studies, including Japanese phase II nivolumab and CheckMate 358, similarly demonstrated enriched responses in PD-L1–positive populations (106, 107).

**Table 3:** Pivotal clinical trials of immunotherapy in cervical cancer by PD-L1 expression status.

Trial	Phase	Population	Regimen	PD-L1 Threshold	ORR (PD-L1+)	ORR (PD-L1-)	PFS HR (95% CI)	OS HR (95% CI)	Reference
KEYNOTE-158	II	Recurrent/metastatic (≥2L)	Pembrolizumab monotherapy	CPS ≥1	14.6% (12/82)	0% (0/16)	NR	NR	(105, 107)
KEYNOTE-826	III	Persistent/recurrent/metastatic (1L)	Pembrolizumab + chemo ± bevacizumab	CPS ≥1	NR	NR	0.62 (0.50–0.77)	0.64 (0.50–0.81)	(103)
				CPS ≥10	NR	NR	0.58 (0.44–0.77)	0.61 (0.44–0.84)	(103)
				CPS <1	NR	NR	0.72 (0.52–1.00)	0.81 (0.55–1.19)	(103)
EMPOWER-Cervical 1	III	Recurrent/metastatic (≥2L)	Cemiplimab vs. chemotherapy	TPS ≥1%	18% (11–28)	11% (4–25)	0.73 (0.58–0.92)	0.70 (0.55–0.89)	(108)
BEATcc	III	Recurrent/metastatic (1L)	Atezolizumab + chemo + bevacizumab	CPS ≥1	NR	NR	0.60 (0.45–0.80)	0.65 (0.46–0.91)	(109)
				CPS <1	NR	NR	0.71 (0.49–1.03)	0.82 (0.54–1.24)	(109)
Nivolumab Japanese phase II	II	Advanced/recurrent (≥2L)	Nivolumab monotherapy	IHC ≥1%	33% (5/15)	0% (0/5)	NR	NR	(110)
CheckMate 358	I/II	Recurrent/metastatic (viral-associated tumors)	Nivolumab ± ipilimumab	TPS ≥1%	26.3%	20.0%	NR	NR	(111)

The optimal PD-L1 scoring method and threshold continue to evolve. Studies have employed CPS (PD-L1+ cells/total viable cells × 100) and TPS (tumor cell percentage), with CPS showing better discriminatory ability. Cutoff choice (CPS ≥1 vs. ≥10) impacts patient selection: higher cutoffs identify populations with greater benefit but exclude patients who might still derive benefit. A meta-analysis of 1,847 patients from four randomized trials found that while treatment effect was larger in CPS ≥1 patients (HR 0.68; 95% CI 0.58–0.79), CPS <1 patients still derived significant benefit (HR 0.79; 95% CI 0.64–0.97), supporting inclusion of PD-L1–negative patients in clinical practice (108). PD-L1 expression is heterogeneous across tumor regions and over time, causing discordance between biopsy and resection specimens in 27% of biopsies, supporting careful timing of testing and consideration of re-sampling at progression (112). HPV status modulates immune contexture; HPV16-positive oropharyngeal tumors show higher CD8+ TIL densities and more frequent PD-L1 positivity than HPV-negative tumors, and combined assessment (e.g., CD8-high/PD-L1-high) is associated with favorable outcomes, suggesting composite biomarkers may outperform PD-L1 alone (113).

**Table 4** provides a comprehensive overview of biomarkers associated with immunotherapy response in cervical cancer. PD-L1 expression by CPS remains the most robustly validated biomarker, with phase III trial evidence supporting its use as a companion diagnostic for pembrolizumab in first-line cervical cancer (104, 108, 114–116). The CPS ≥1 cutoff identifies 85–90% of cervical cancers as PD-L1 positive, with patients deriving significant benefit from pembrolizumab-based therapy (PFS HR 0.62; 95% CI 0.50–0.77), though benefit is also observed in CPS <1 patients (PFS HR 0.72; 95% CI 0.52–1.00) (104, 108). The CPS ≥10 cutoff identifies a smaller subset (45–55%) with even greater benefit (PFS HR 0.58; 95% CI 0.44–0.77) and may identify “super-responder” populations (103, 104). TPS-based scoring is less discriminative due to its failure to capture immune cell PD-L1 expression (117–119). TIL density correlates with improved outcomes in retrospective studies but lacks standardization for clinical use (120, 121). TMB shows moderate association with response, though cervical-specific data remain limited (122–128). HPV genotype data are inconclusive and not clinically actionable (126, 129–134). Immune gene signatures and APOBEC mutational signatures represent emerging investigational biomarkers (135, 136, 139–143). MSI-H/dMMR status, while rare (2–5%), confers high response rates and warrants routine testing per NCCN guidelines (125, 137, 138). Current evidence supports mandatory PD-L1 CPS testing for all advanced cervical cancer patients considered for immunotherapy, with MSI-H/dMMR testing as an additional standard; other biomarkers remain investigational (108, 114–116).

**Table 4:** Predictive biomarkers for immunotherapy response in cervical cancer.

Biomarker	Prevalence in cervical cancer	Association with ICI response	Evidence level	Detection method	Key findings	Clinical utility	References
PD-L1 CPS ≥1	85–90%	Positive	Phase III trials	Immunohistochemistry (IHC) using 22C3, 28-8, SP142, or SP263 assays; CPS = (PD-L1+ tumor cells + immune cells)/total viable tumor cells × 100	KEYNOTE-826: PFS HR 0.62 (0.50–0.77) for CPS ≥1 vs. 0.72 (0.52–1.00) for CPS <1. EMPOWER-Cervical 1: ORR 18% (CPS ≥1) vs. 11% (CPS <1). BEATcc: PFS HR 0.60 (0.45–0.80) for CPS ≥1.	FDA-approved companion diagnostic for pembrolizumab in cervical cancer; guides first-line treatment decisions	(104, 108, 114–116)
PD-L1 CPS ≥10	45–55%	Strong positive	Retrospective analyses	IHC (same assays as above); higher cutoff for greater specificity	KEYNOTE-826: PFS HR 0.58 (0.44–0.77) for CPS ≥10. Greater magnitude of benefit than CPS ≥1. Identifies population with highest likelihood of response.	Identifies “super-responder” population; may be considered for treatment prioritization in resource-limited settings	(103, 104)
PD-L1 TPS ≥1%	20–45% (lower than CPS)	Positive (benefit seen but less discriminative than CPS)	Phase III trial context (EMPOWER-Cervical 1)	IHC; TPS = (PD-L1+ tumor cells)/total tumor cells × 100	EMPOWER-Cervical 1: PFS HR 0.73 (0.58–0.92) for TPS ≥1%	Alternative scoring; CPS generally preferred in cervical cancer due to immune cell contribution	(117–119)
Tumor-Infiltrating Lymphocyte (TIL) Density	Highly variable (10–80%)	Positive	Retrospective cohort studies	H&E staining or IHC for CD3+, CD4+, CD8+ T cells; stromal vs. intratumoral assessment	High CD8+ TIL density correlates with improved PFS/OS and HPV-driven immune activation; retrospective signals for PD-1/PD-L1 response	Not standardized for clinical use; limited by inter-observer variability and lack of prospective validation	(120, 121)
Tumor Mutational Burden (TMB-H)	12–18% (≥10 mut/Mb). 3–5% (≥20 mut/Mb).	Moderate	Exploratory/Retrospective	Next-generation sequencing (NGS) using validated panels (e.g., FoundationOne, MSK-IMPACT)	KEYNOTE-158: ORR 29% in TMB-H (≥10 mut/Mb) vs. 6% in TMB-low across multiple tumor types; cervical-specific data limited. TMB-H linked to higher neoantigen load and immune recognition; predictive strength varies by tumor immune microenvironment.	Not approved as standalone biomarker in cervical cancer; may complement PD-L1 testing in selected cases	(122–128)
HPV Genotype	HPV16: 60–70%. HPV18: 10–15%. Other HR-HPV: 15–20%.	Inconclusive	Preclinical/Early translational	HPV DNA testing (PCR), HPV RNA *in situ* hybridization, or NGS-based viral detection	HPV16-positive tumors may have higher PD-L1 expression than other HPV genotypes. HPV E6/E7 oncoproteins modulate immune microenvironment. Some studies suggest differential response by HPV genotype, but data inconsistent.	Not clinically actionable; insufficient evidence to guide treatment selection	(126, 129–134)
Immune Gene Signatures	Variable	Positive	Exploratory	RNA sequencing or Nanostring-based immune profiling	Interferon-gamma/T-cell–inflamed gene expression profile correlates with pembrolizumab response; chemokines CXCL9/CXCL10 and CD8A reflect T-cell–inflamed status linked to benefit	Investigational only; not routinely available in clinical practice	(135, 136)
MSI-H/dMMR	2–5%	Positive (rare)	Phase II basket trials	IHC for MMR proteins (MLH1, MSH2, MSH6, PMS2) or PCR for MSI	Tumor-agnostic FDA approval for pembrolizumab in MSI-H/dMMR solid tumors. Rare in cervical cancer but associated with high response rates when present. KEYNOTE-158: 3/5 MSI-H cervical cancer patients responded.	Routine testing recommended per NCCN guidelines; high impact for rare subset	(125, 137, 138)
APOBEC Mutational Signature	~40–60% in HPV-associated cervical cancer cohorts	Emerging; linked to higher TMB and immune activation, suggesting potential ICI benefit	Translational (retrospective and correlative)	Whole-exome or targeted NGS; mutational signature analysis	APOBEC3-mediated mutagenesis is common in cervical cancer and enriched in PD-L1–positive tumors. HPV-driven tumors show APOBEC-linked driver mutations (e.g., *PIK3CA*) and immune-associated signatures.	Investigational; no prospective validation	(139–143)

1L, first-line; 2L, second-line; CI, confidence interval; CPS, combined positive score; HR, hazard ratio; IHC, immunohistochemistry; NR, not reported; ORR, objective response rate; OS, overall survival; PFS, progression-free survival; TPS, tumor proportion score.

Ongoing studies are investigating whether these additional biomarkers can refine patient selection, particularly for PD-L1-negative patients who currently derive only modest benefit from ICI therapy. These include composite genomic/immune signatures (e.g., TMB+GEP, MPS+TIDE), blood-based markers (ctDNA, TCR repertoire), and host factors (microbiome, HLA diversity), aiming to outperform single-analyte PD-L1 by integrating tumor genomics and the tumor microenvironment (144). Early data also explore peripheral indices such as prognostic nutritional index to stratify outcomes in PD-L1-negative, microsatellite-stable disease (145).

Despite this success, approximately 50% of gynecologic cancer patients remain refractory to ICIs, prompting strategies that prime or enhance antitumor immunity through combinations such as PD-1/PD-L1 plus CTLA-4 blockade, PARP inhibitors, VEGF-targeted agents, and chemotherapy, alongside novel approaches like cancer vaccines and adoptive cell therapies to re-sensitize tumors to checkpoint blockade (72, 146). The effectiveness and durability of these combinations will depend on identifying predictive biomarkers (e.g., MMR/MSI status, TMB, PD-L1 expression, T cell–inflamed gene signatures) to match patients with the most rational regimen and mitigate added toxicity (72, 144, 146–150).

### 4.3 The Renaissance of cytotoxic payloads: antibody-drug conjugates

ADCs represent a significant advance in targeted therapy by combining the tumor-specific targeting of monoclonal antibodies with highly potent cytotoxic payloads, thereby minimizing off-target toxicity and widening the therapeutic window compared to traditional chemotherapy (151–153).

Three ADCs have established new standards of care in specific gynecologic cancers. Tisotumab vedotin targets tissue factor (TF), which is highly expressed in cervical cancer. Its accelerated FDA approval was based on the phase II innovaTV-204/GOG-3023/ENGOT-cx6 trial, which demonstrated a confirmed objective response rate of 24% (95% CI 16-33%) in heavily pretreated recurrent/metastatic cervical cancer, with a median duration of response of 8.3 months (95% CI 4.2–not reached) (154, 155). Mirvetuximab soravtansine targets folate receptor alpha (FRα) in platinum-resistant ovarian cancer. The pivotal phase III MIRASOL trial established its superiority over investigator’s choice chemotherapy in patients with FRα-high tumors (defined as ≥75% of cells with ≥2+ staining intensity), demonstrating significantly improved progression-free survival (HR 0.65 [95% CI 0.52–0.81]; *P* < 0.0001) and overall survival (HR 0.67 [95% CI 0.50–0.89]; *P* = 0.005), leading to its approval as a new biomarker-defined standard of care (156). For HER2-expressing tumors, trastuzumab deruxtecan (T-DXd) shows significant promise. Real-world data reports a median progression-free survival of 5.4 months (95% CI 0.8–9.8 months) and a 50% partial response rate in heavily pretreated patients with HER2-expressing gynecologic malignancies (157). This is clinically pertinent, as 59.5% of endometrial cancers express HER2, with 30.5% showing clinically actionable strong (2+/3+) expression a feature associated with high-risk disease, advanced stage, and aggressive histology (158).

Next−generation ADCs are expanding options in gynecologic cancers. Upifitamab rilsodotin (UpRi), a NaPi2b (SLC34A2)–directed ADC, yielded an objective response rate (ORR) of ~34% in platinum−resistant ovarian cancer with high NaPi2b expression in phase II, supporting NaPi2b as a therapeutically targetable antigen and spurring biomarker cutoff refinement, though pulmonary (pneumonitis) and ocular toxicities have tempered development momentum (153, 159). Parallel efforts with novel HER2−directed ADCs show encouraging signals: early−phase studies of trastuzumab duocarmazine (SYD985) and disitamab vedotin (RC48) report preliminary ORRs around 25–40% in heavily pretreated, HER2−expressing gynecologic cohorts, with toxicity generally manageable and characterized by class−related ocular events and neuropathy; RC48 combinations have demonstrated activity across solid tumors and included endometrial cancer responders in phase I expansion (153, 160). Platform innovations such as dual−payload designs and conditionally activated ADCs aim to improve therapeutic index via tumor−selective activation and resistance mitigation, a direction highlighted in recent gynecologic oncology ADC overviews (153). Collectively, these data underscore the importance of antigen selection (for example, NaPi2b) and HER2 expression stratification to optimize benefit−risk in gynecologic settings while newer architectures seek greater tumor specificity (153, 159).

Combination ADC–immunotherapy approaches are gaining momentum. In FRα-positive, platinum-resistant ovarian cancer, mirvetuximab soravtansine plus pembrolizumab showed meaningful activity in phase I/II testing, with an objective response rate (ORR) of 31% (95% CI 19–45) and median duration of response 8.0 months, suggesting benefit in heavily pretreated populations and supporting the concept that ADC-induced immunogenic cell death may prime synergy with checkpoint blockade (161). For context, single-agent mirvetuximab in high FRα expression achieved ORR 42.3% and improved progression-free survival (5.62 vs 3.98 months) and overall survival (16.46 vs 12.75 months) versus chemotherapy in the phase 3 MIRASOL trial, underscoring the ADC’s intrinsic potency to which immunotherapy might add complementary benefit (162).

The literature confirms that these approved ADCs deliver promising objective response rates with manageable toxicity profiles (154). The field is rapidly evolving, with multiple investigational ADCs showing preliminary efficacy in clinical development (154). Significant technological advances, including novel linker chemistry, more potent payloads, and refined antibody engineering in newer-generation agents, are driving improvements in efficacy and safety (163). Future therapeutic directions emphasize next-generation platform innovations and rational combination strategies to overcome resistance, though specific quantitative data validating these advanced approaches requires further clinical research (154, 163).

### 4.4 Targeting oncogenic signaling pathways: beyond single gene alterations

The PI3K/AKT/mTOR pathway represents a critical and frequently dysregulated signaling axis in gynecologic cancers, activated through common genetic alterations such as *PIK3CA* mutations and *PTEN* loss, as well as upstream growth factor receptor signaling (164, 165). This pathway integrates diverse mitogenic stimuli to control essential cellular processes like proliferation, survival, and metabolism, making it a compelling therapeutic target (165).

Clinically, agents targeting this pathway, particularly AKT inhibitors, have demonstrated promising but variable anti-tumor activity, often limited by on-target toxicities and the emergence of resistance (166). Biomarker-driven patient selection is crucial. Preclinical evidence suggests that *PTEN* loss or *PIK3CA* mutations may predict sensitivity to PI3K/AKT/mTOR inhibition. Conversely, co-occurring alterations such as *KRAS* mutations may confer primary resistance, indicating a need for rational combination strategies, potentially with inhibitors of parallel pathways like RAS/RAF/MEK (165).

Isoform-selective PI3K inhibitors have improved therapeutic windows over pan-*PI3K* agents by limiting off−target toxicities and enabling more precise pathway blockade, exemplified by the α−selective inhibitor alpelisib’s approval in *PIK3CA*−mutated HR−positive breast cancer and its expanding evaluation in gynecologic malignancies (167). In breast cancer, α−selective targeting roughly doubled progression−free survival versus placebo when combined with fulvestrant (11.0 vs 5.7 months; HR 0.65), while grade ≥3 hyperglycemia and rash were the most frequent adverse events (36.6% and 9.9%) (167). Across trials and meta-analyses, hyperglycemia is the predominant toxicity (all−grade ~59%; grade 3/4 ~28–37%), with grade ≥3 rash ~9–10%, generally manageable with dose modification and supportive care (168, 169). Mechanistic and correlative data consistently implicate *PTEN* loss as a resistance mechanism to PI3Kα inhibition, underscoring the need for comprehensive genomic profiling when selecting patients for isoform−specific PI3K therapy (170).

The AKT inhibitor capivasertib has demonstrated a more favorable toxicity profile than pan-PI3K inhibitors and is being investigated in multiple gynecologic cancer contexts. The phase II OCTOPUS trial (NCT04729387) is evaluating capivasertib in combination with olaparib in ovarian cancer, leveraging preclinical evidence of synthetic lethality between AKT inhibition and PARP inhibition in PTEN-deficient tumors; early data suggest this combination may overcome PARP resistance in a subset of patients (171). Similarly, the dual PI3K/mTOR inhibitor gedatolisib is being evaluated in combination with palbociclib in endometrial cancer (NCT03065062), representing a strategy to simultaneously target the PI3K/AKT/mTOR and cell cycle pathways (172).

Emerging resistance mechanisms to PI3K/AKT/mTOR inhibitors are increasingly mapped by serial tissue and liquid biopsies, revealing convergent escape via feedback activation of receptor tyrosine kinases (RTKs) such as IGF1R and HER-family receptors, AKT rebound through Skp2–IGF1R–PDK1–mTORC2 axes, and compensatory MAPK pathway upregulation, alongside on-target alterations including PTEN loss, secondary PIK3CA mutations that remodel the inhibitor binding pocket, and PIK3CA amplification (170, 173–176). Additional mechanisms include HIF/PIM signaling and ERK/MAPK cross-talk, each restoring downstream survival signaling despite pathway blockade (175, 176). Clinically, approximately half of patients progressing on PI3Kα inhibitors acquire PI3K-pathway genomic events (e.g., PTEN loss, AKT1 activation), and secondary PIK3CA resistance mutations have been identified by serial liquid biopsies; some of these are suppressible by emerging allosteric, pan-mutant PI3Kα inhibitors, underscoring the value of genotype-directed sequencing strategies at progression (170).

Rational combination strategies are advancing, including dual PI3K/MAPK targeting based on reciprocal compensatory activation and preclinical synergy that suppresses outgrowth at lower doses. Early trials and translational studies emphasize that overlapping on-target toxicities (e.g., hyperglycemia, rash, diarrhea) demand careful dose selection, intermittent scheduling, and biomarker-guided patient selection to preserve therapeutic index (175, 177).

Despite these challenges, numerous inhibitors targeting various nodes of the PI3K/AKT/mTOR cascade continue to be evaluated in phase II/III clinical trials. However, definitive evidence of their clinical utility and the establishment of robust predictive biomarkers require validation in larger, confirmatory studies (166).

## 5 The inevitable adversary: mechanisms of therapeutic resistance

**Figure 2:**
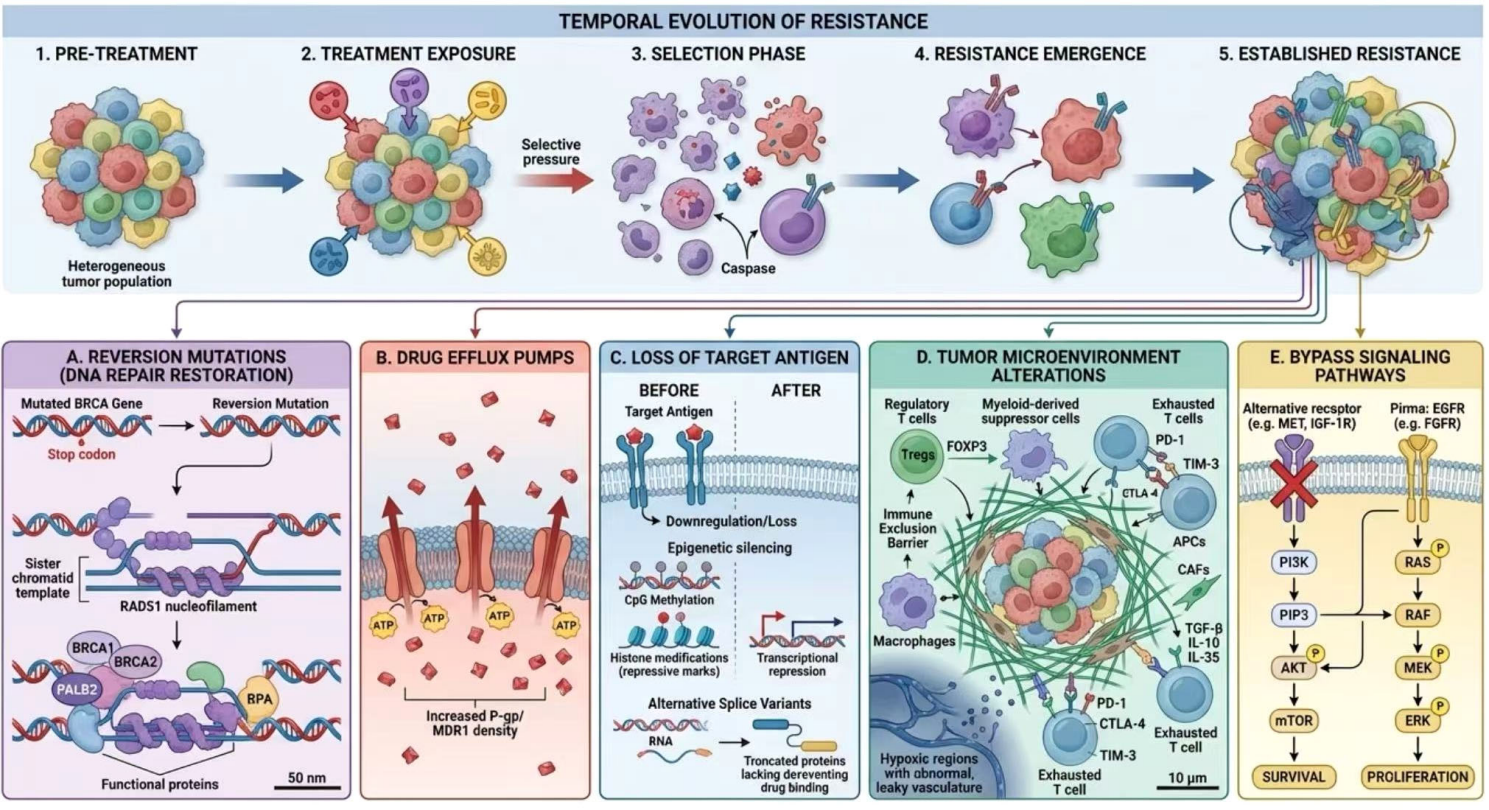
Mechanisms of acquired resistance to targeted therapies in gynecologic cancers. A conceptual model illustrating the evolution of resistance under therapeutic pressure. The figure details five principal mechanisms: **(A)** Reversion mutations restoring homologous recombination function, **(B)** Upregulation of drug efflux pumps, **(C)** Loss of target antigen expression, **(D)** Alterations in the tumor microenvironment promoting immune exclusion/suppression, and **(E)** Activation of bypass signaling pathways (e.g., PI3K/AKT/mTOR, MAPK/ERK). The model emphasizes that multiple mechanisms frequently coexist, necessitating combination therapeutic strategies.

The initial success of targeted therapies is consistently tempered by the emergence of acquired resistance, a consequence of evolutionary selection within molecularly diverse and dynamic tumor ecosystems (178). Under the strong selective pressure of treatment, pre-existing resistant subclones can expand, or new resistance mutations can emerge through genomic instability (178). Resistance mechanisms are multifaceted, encompassing both intrinsic tumor cell factors (e.g., genetic mutations, pathway activation, epigenetic plasticity) and extrinsic alterations in the tumor microenvironment that create protective niches (178, 179). Key resistance pathways for major drug classes are summarized in **Figure 2**.

PARP Inhibitor Resistance: Despite high initial efficacy, over 40% of patients with *BRCA1/2*-deficient tumors exhibit primary or acquired resistance to PARP inhibitors (PARPi) (180). Documented mechanisms include restoration of homologous recombination repair (28% of cases), stabilization of replication forks (34%), upregulation of parallel survival pathways (41%), target loss (10%), and increased drug efflux (3%) (181). A predominant genetic mechanism is the restoration of HR proficiency via secondary *BRCA1/2* reversion mutations or epigenetic reversal of BRCA1 promoter methylation (182).

Immunotherapy Resistance: Resistance to immune checkpoint inhibitors (ICIs) can also develop in MSI-H/dMMR tumors, despite high tumor mutational burden and PD-L1 expression (183). Mechanisms involve tumor-intrinsic adaptations, such as loss of antigen presentation (*B2M* mutations) and defects in interferon-gamma signaling (*JAK1/2, STAT1* mutations), as well as microenvironmental remodeling leading to T-cell exclusion, enrichment of immunosuppressive cells, and upregulation of alternative checkpoints (e.g., LAG-3, TIM-3) (183). Unique anatomical sites, such as peritoneal ascites, can further foster resistance by creating immunoprivileged environments (184).

ADC Resistance: Resistance to ADCs can involve any component of their mechanism: loss or downregulation of the target antigen, altered internalization or intracellular trafficking, efflux of the cytotoxic payload via ABC transporters, and activation of downstream survival or bypass pathways (185, 186). Specific payload-related resistance mechanisms have been identified, including loss of *SLC46A3* expression, a lysosomal transporter required for the activation of ADCs bearing certain payloads like DM1 and SG3376 (187, 188).

Recent single-cell and spatial transcriptomics have uncovered non-genetic resistance programs in ovarian cancer, especially under PARP inhibitor and chemotherapy pressure. Single-cell RNA-seq of pre- and post-PARP inhibitor models shows emergence of drug−tolerant persister states with slow−cycling, stem−like phenotypes marked by developmental pathway activation (WNT, NOTCH) and epigenetic reprogramming, providing a reservoir for later genetic resistance; epigenetic regulators (e.g., EZH2, CREBBP, CARM1) and WNT components (e.g., FZD10) have been linked to progression and PARP inhibitor resistance signatures, supporting a model where chromatin−driven plasticity enables persistence before mutation fixation (189). Complementary single−cell chromatin accessibility profiling of high−grade serous ovarian carcinoma metastases demonstrates that chemotherapy selects for pre−existing subpopulations with accessible enhancers governing anti−apoptotic, DNA repair, and stress−response programs (e.g., motifs for TP53/TP63/TWIST1), while losing proliferative E2F programs defining residual, low−proliferative, chemoresistant cells and nominating nuclear receptors (RORα, NR2F6, HNF4G) as candidate drivers of these states; these findings directly support the concept that distinct chromatin architectures predate therapy and are enriched by treatment to seed recurrence (190).

Metabolic reprogramming is a key non−genetic resistance mechanism. In BRCA−mutant ovarian cancer with acquired PARP inhibitor (PARPi) resistance, stable−isotope and metabolomics studies show a shift toward increased oxidative phosphorylation (OXPHOS) and glutamine dependence; targeting glutaminase with CB−839 restores PARPi sensitivity and shows synergy in resistant xenografts, consistent with HR−defective tumors relying on oxidative metabolism and glutaminolysis to sustain NAD+/ATP for DNA repair and survival (191, 192). Preclinical work demonstrates that GLS−high ovarian cancer cells are intrinsically chemoresistant and that CB−839 induces reactive oxygen species and replication stress, rendering tumors vulnerable to olaparib and prolonging survival in mouse models; this rationale underpins ongoing clinical evaluation (e.g., NCT03875313) (191). Similarly, tumors resistant to immunotherapy exhibit metabolic profiles characterized by heightened glycolysis and lactate production, promoting an acidic, immunosuppressive microenvironment that impairs effector T−cell function; driving glycolysis and lactate can repress homologous recombination gene expression via histone deacetylation and create “BRCAness,” which in turn modulates sensitivity to PARPi and immune pressure, highlighting metabolism–DNA repair–immunity crosstalk (193). Together, these data support exploiting metabolic liabilities OXPHOS/glutamine in PARPi−resistant BRCA−mutant ovarian cancer and glycolysis/lactate programs in immunotherapy resistance to resensitize tumors with rational combinations such as PARPi plus glutaminase inhibition (191–193).

**Table 5** summarizes emerging non-genetic resistance mechanisms in gynecologic cancers. Drug-tolerant persister states (WNT/NOTCH/YAP/TAZ activation) are targetable with HDAC/WNT/NOTCH inhibitors (194–196). Metabolic reprogramming (OXPHOS/glutamine dependence) in PARPi-resistant ovarian cancer creates vulnerability to glutaminase inhibitors (CB-839) (191, 197). Epigenetic plasticity (BET/EZH2/HDAC) enables adaptive resistance via chromatin remodeling (198, 199). Alternative splicing generates DNA repair gene variants targetable by spliceosome modulators (200–204). Autophagy provides cytoprotection, with hydroxychloroquine under investigation (205, 206). Senescence escape is targetable with senolytics (navitoclax) (207, 208). TME remodeling (CAF/ECM/immune exclusion) creates protective niches (209–215). Mitochondrial dynamics (DRP1) represent an emerging vulnerability (216, 217). These mechanisms complement genetic resistance and offer novel therapeutic opportunities.

**Table 5:** Emerging non-genetic mechanisms of therapeutic resistance and therapeutic vulnerabilities.

Mechanism	Key findings	Cancer type(s)	Therapeutic vulnerability	Investigational agents	Clinical trial status	Biomarkers	References
Drug-tolerant persister states	Activation of developmental pathways (WNT, NOTCH, YAP/TAZ).Stem-like phenotype with slow-cycling characteristics.Enhanced DNA repair capacity.Epigenetic reprogramming with distinct chromatin accessibility profiles.Pre-existing subpopulations enriched by therapy.	Ovarian, Endometrial	HDAC inhibitors WNT inhibitors NOTCH inhibitors CDK9 inhibitors	Panobinostat (HDAC).Entinostat (HDAC).LGK974 (WNT).Crenigacestat (NOTCH).Alvocidib (CDK9).	Preclinical.	CD133, ALDH1 (stemness markers).	(194–196)
Metabolic reprogramming	Fatty acid oxidation dependence.	Ovarian (PARPi-resistant), Cervical	Glutaminase inhibitors OXPHOS inhibitors Glycolysis inhibitors Fatty acid synthesis inhibitors	TVB-2640 (FASN inhibitor).	Phase II NCT01528046 (Metformin).	FDG-PET avidity.	(191, 197)
Epigenetic plasticity	EZH2-mediated polycomb repression.	Ovarian, Endometrial	BET inhibitors EZH2 inhibitors HDAC inhibitors DNMT inhibitors	Azacitidine, Decitabine (DNMT).	Phase II NCT02316340 (HDAC).	Methylation signatures.	(198, 199)
Alternative splicing	Aberrant exon inclusion/exclusion.	Ovarian, Cervical	Spliceosome modulators SF3B1 inhibitors CLK inhibitors	TG003 (CLK).	Phase I NCT05014646 (Cirtuvivint).	SF3B1 expression.	(200–204)
Autophagy induction	Cytoprotective response to therapy.	Platinum-resistant ovarian	Autophagy inhibitors Lysosomal inhibitors mTOR activators	Spautin-1 (ULK1 inhibitor).	Phase I NCT04523857 (Lys05).	TFEB nuclear localization.	(205, 206)
Senescence escape	p16, p21, p53 dynamics.	Ovarian, Endometrial	Senolytics Senomorphics CDK4/6 inhibitors	Palbociclib (CDK4/6).	Phase II NCT03403270 (Palbociclib).	SASP factors (IL-6, IL-8).	(207, 208)
Tumor microenvironment remodeling	Exosome-mediated transfer of resistance traits.	Ovarian (ascites), Cervical	CAF inhibitors HIF2α inhibitors Immune checkpoint combinations ECM modulators	Losartan (AT1R antagonist).	Phase II NCT02715804 (PEGPH20).	ECM stiffness (elastography).	(209–215)
Mitochondrial dynamics	ROS signaling adaptation.	Platinum-resistant ovarian	Mitochondrial dynamics modulators DRP1 inhibitors Mitochondrial translation inhibitors	Phenformin.	Phase I NCT03291938 (IACS-010759).	ROS levels.	(216, 217)

APOBEC, apolipoprotein B mRNA editing enzyme catalytic polypeptide-like; CI, confidence interval; CPS, combined positive score; dMMR, deficient mismatch repair; FDA, Food and Drug Administration; H&E, hematoxylin and eosin; HPV, human papillomavirus; HR, hazard ratio; HR-HPV, high-risk human papillomavirus; ICI, immune checkpoint inhibitor; IHC, immunohistochemistry; MSI-H, microsatellite instability-high; NCCN, National Comprehensive Cancer Network; NGS, next-generation sequencing; ORR, objective response rate; OS, overall survival; PCR, polymerase chain reaction; PD-1, programmed cell death protein 1; PD-L1, programmed death-ligand 1; PFS, progression-free survival; TIL, tumor-infiltrating lymphocyte; TMB, tumor mutational burden; TMB-H, tumor mutational burden-high; TPS, tumor proportion score.

Emerging evidence indicates that resistance arises through both fixed genetic alterations and reversible, non-genetic cell-state transitions, arguing for therapies that jointly suppress resistant clones while exploiting fitness trade-offs and collateral sensitivities (218). Mathematical and evolutionary models predict that maintaining competition between sensitive and resistant populations via non−maximal, adaptive or intermittent dosing can prolong control compared with continuous maximum−tolerated dose, even when resistance carries no fitness cost (219).

Intermittent/adaptive schedules vs. continuous dosing: Across theoretical analyses and simulations, strategies that modulate dose to contain rather than eradicate sensitive cells can delay clonal takeover and extend time to progression, whereas more aggressive constant dosing accelerates selection for resistant phenotypes (219). By steering clonal composition toward states with collateral vulnerabilities, evolutionary “herding” enables sequential regimens that regain efficacy when switching drugs (218). Together, these data support exploring evolutionarily informed dosing and steering to prevent or delay resistance, instead of relying solely on continuous maximum−tolerated approaches (218, 219).

Beyond these class-specific mechanisms, a broader understanding of tumor evolution is critical. Evolutionary convergence analyses highlight non-genetic adaptations, such as metabolic reprogramming and epigenetic plasticity, which also drive resistance and may require different therapeutic approaches (178, 220). Mathematical modeling suggests that combination therapies targeting multiple vulnerabilities simultaneously may raise the evolutionary barrier and delay or prevent resistance, though optimal strategies remain an active area of investigation (178).

### 5.1 Heterogeneity and resistance: spatial and temporal dimensions

Tumor heterogeneity functions across multiple interconnected spatial and temporal scales, fundamentally driving therapeutic resistance through distinct yet overlapping mechanisms. Intratumoral heterogeneity, characterized by profound genetic and phenotypic diversity within a single tumor, creates a spatial mosaic of subclones with varying drug sensitivities. Resistant cell populations may exist initially as minor subclones or minimal residual disease but can dominate following the selective pressure of treatment (221). This spatial complexity creates a critical sampling bias, where biopsies from single tumor regions may fail to detect resistant clones present elsewhere, leading to an underestimation of resistance mechanisms and treatment failure (222).

Temporal heterogeneity, or continuous tumor evolution, compounds this challenge. Pre-existing resistant cell populations can persist from diagnosis, matching the genomic and transcriptomic profiles of resistant populations that emerge post-treatment. This is exemplified in prostate cancer, where certain tumor cell clusters present prior to androgen deprivation therapy share gene expression identities with resistant clusters that arise after treatment (223). This evolution is driven by genomic instability, epigenetic plasticity, and heterogeneous microenvironmental pressures (222).

Addressing this complexity requires a fundamental shift in therapeutic strategy. A single-agent approach often selects for pre-existing resistant clones. Consequently, the field is moving towards tailored combination strategies designed to target multiple core oncogenic drivers and resistance pathways simultaneously (222). Emerging technologies such as liquid biopsies and single-cell sequencing are critical for mapping these multiscale heterogeneity patterns. They enable the identification of targetable drivers in minimal residual disease and resistant subclones, informing the development of preemptive combinatorial therapies aimed at eradicating both primary and resistant tumor populations from the outset (221).

### 5.2 The microenvironment’s role in fostering resistance

The tumor microenvironment (TME) functions as an active and dynamic ecosystem that fosters therapeutic resistance through complex, interconnected mechanisms beyond the cancer cell itself. The extracellular matrix can create dense physical barriers that impede drug penetration, while cancer-associated fibroblasts (CAFs) promote resistance by secreting a protective milieu of cytokines, chemokines, and growth factors that enhance cancer cell survival (224).

A critical microenvironmental condition is the establishment of hypoxic and nutrient-deprived niches. These selective pressures not only drive the evolution of resistant tumor clones but also alter local drug metabolism and distribution, collectively impairing treatment efficacy (225). The TME further orchestrates an immunosuppressive network where infiltrating immune cells are co-opted into phenotypes (e.g., regulatory T cells, tumor-associated macrophages) that actively dampen cytotoxic anti-tumor immunity, facilitating immune escape (225, 226).

This enhanced communication between the tumor and its surrounding stroma creates complex and adaptable resistance patterns. As exemplified in hepatocellular carcinoma, the collective interaction of CAFs, tumor vasculature, immune cells, and secreted factors like exosomes underpins diverse resistance mechanisms, making the TME a particularly challenging therapeutic target (226).

To decode these interactions, multi-omics approaches have become essential. By integrating genomic, transcriptomic, and metabolomic data, researchers can map the intricate molecular networks between tumor cells and their microenvironment, identifying novel therapeutic vulnerabilities and strategies to overcome this critical barrier to durable treatment response (225).

## 6 Future directions: novel diagnostics and adaptive trial paradigms

To overcome resistance and extend the benefits of precision oncology, innovations in diagnostics and clinical research are required. Liquid biopsy analysis of circulating tumor DNA (ctDNA) provides a noninvasive tool to capture tumor heterogeneity, monitor clonal evolution in real time, and guide treatment strategies after resistance emerges (227). Concurrently, the development of next-generation therapeutics including bispecific antibodies, ADCs, and RNA-based therapies promises to expand treatment options in challenging clinical scenarios (227).

### 6.1 Liquid biopsy and longitudinal monitoring

The analysis of circulating tumor DNA (ctDNA) and other blood-based biomarkers collectively termed liquid biopsy offers a minimally invasive approach to precision oncology by enabling identification of actionable mutations when tumor tissue is insufficient or inaccessible, improving turnaround time and capturing spatial/temporal heterogeneity for targeted therapy selection; by detecting minimal residual disease (MRD) after curative-intent surgery or chemoradiation, where ctDNA positivity can reveal microscopic disease before imaging and inform adjuvant treatment escalation/de-escalation; and by providing real-time longitudinal monitoring to track tumor evolution, quantify response, and uncover emerging resistance mechanisms during targeted or immunotherapy, thus guiding timely therapeutic adjustments (228, 229).

A principal application is the dynamic surveillance of resistance mechanisms. Longitudinal ctDNA profiling can detect specific genetic drivers of resistance, such as *BRCA1/2* reversion mutations, found in 60% of patients developing resistance to HRD-targeted therapy, often months before clinical progression (59). Serial analysis reveals the clonal complexity of resistance, exposing multiple co-occurring pathways including homologous recombination repair restoration, replication fork stabilization, and upregulation of survival pathways with mutational heterogeneity increasing post-progression in the majority of patients (181).

Emerging technologies are expanding the scope and sensitivity of liquid biopsy applications. Methylation-based circulating tumor DNA analysis has shown particular promise for early detection and minimal residual disease monitoring. The prospective, multi-center EPIRM-OC study (NCT04853784) evaluated a liquid biopsy panel combining mutation and methylation markers in 587 women with adnexal masses, achieving 89% sensitivity and 93% specificity for ovarian cancer detection, with performance superior to CA-125 alone (230). For endometrial cancer, a methylation-based ctDNA assay detected 71% of early-stage (I–II) cancers in a case-control study, suggesting potential for screening in high-risk populations (231).

Fragmentomics the analysis of cell-free DNA fragmentation patterns represents another innovative approach that does not require prior knowledge of tumor mutations. Bolck and colleagues (2024) demonstrated that genome-wide fragmentation profiles could detect ovarian cancer with 82% sensitivity at 94% specificity in a validation cohort, with fragment patterns correlating with tumor burden and treatment response (230). Similarly, nucleosome positioning maps derived from cfDNA sequencing have been shown to infer tumor-of-origin and gene expression programs, potentially enabling non-invasive transcriptional profiling (232).

**Table 6** summarizes emerging liquid biopsy technologies in gynecologic cancers. Methylation-based ctDNA assays achieve 89%/93% sensitivity/specificity for ovarian and 71% for early-stage endometrial cancer detection (233–239). Fragmentomics provides 82%/94% sensitivity/specificity for ovarian cancer without prior mutation knowledge (230, 240–243). Phased variant enrichment enables ultra-sensitive MRD detection at 0.001% VAF, detecting relapse months before imaging (244, 245). Simultaneous mutation/methylation panels achieve 99% specificity across five cancer types (246, 247). Tumor-educated platelets and exosomal miRNA offer complementary RNA-based approaches (248–254). Multi-analyte strategies combining ctDNA with proteins (CA-125, HE4) boost sensitivity to 94% (255, 256). Phosphorylated protein and AI-enhanced fragmentomics represent emerging frontiers (257–259). Together, these technologies are transforming early detection, MRD monitoring, and resistance surveillance.

**Table 6:** Emerging liquid biopsy technologies in gynecologic cancers.

Technology	Application	Cancer type(s)	Performance metrics	Limit of detection	Sample type	Advantages	Limitations	Clinical utility	Development phase	References
Methylation-based ctDNA	Early detection	Ovarian	89% sensitivity, 93% specificity	0.01–0.1%	Plasma	Tissue-of-origin inference possible.	Assay standardization challenges.	Adjunct to CA-125.	Validated in prospective cohort (EPIRM-OC)	(233–237)
Methylation-based ctDNA	Early detection	Endometrial	71% sensitivity (stage I–II)	0.05–0.1%	Plasma, Pap brushings	Compatible with liquid-based cytology.	Uterine-specific markers needed.	Triage for abnormal bleeding.	Case-control validation; prospective trials ongoing	(238, 239)
Fragmentomics	Ovarian cancer detection	Ovarian	82% sensitivity, 94% specificity	0.1%	Plasma	Cost-effective.	Influenced by pre-analytical variables.	Complement to imaging.	Retrospective validation; prospective studies ongoing	(230, 240–243)
Phased variant enrichment	MRD detection	Ovarian, Endometrial	Detection limit 0.001% VAF	0.001% (10 parts per million)	Plasma	Personalized assay design.	Turnaround time 2–3 weeks.	Early relapse detection.	Clinical validation; prospective trials (STIC-2)	(244, 245)
Simultaneous mutation/methylation	Multi-cancer early detection	Ovarian, Endometrial, Cervical	5 cancer types at 99% specificity	0.01–0.1%	Plasma	Single workflow.	Higher cost.	Risk stratification.	Multi-cancer early detection (MCED) trials	(246, 247)
Tumor-educated platelets (TEPs)	Early detection, diagnosis	Ovarian	88% accuracy distinguishing benign vs. malignant	N/A	Whole blood	Minimal processing.	Limited validation.	Adjunct diagnostic tool.	Proof-of-concept studies	(248, 249)
Exosomal miRNA	Early detection, monitoring	Ovarian	85% sensitivity, 87% specificity	0.1–1%	Plasma, serum	Reflects TME.	Low RNA yield.	Resistance prediction.	Exploratory; multicenter validation	(250–254)
ctDNA methylation + protein	Multi-analyte early detection	Ovarian	92% sensitivity, 98% specificity (stage III/IV)	0.01%	Plasma	Improved specificity.	Costly.	High-performance screening.	Prospective cohort (STRIVE)	(255, 256)
Phosphorylated cell-free proteins	Early detection	Ovarian	86% sensitivity, 95% specificity	pM range	Plasma	Rapid assay.	Limited multiplexing.	Screening adjunct.	Early validation	(257, 258)
AI-enhanced fragmentomics	Early detection, tumor typing	Ovarian, Endometrial	91% sensitivity, 96% specificity for ovarian	0.05%	Plasma	Scalable.	Requires large training sets.	Triage tool.	Retrospective training; prospective validation ongoing	(230, 259)

AI, artificial intelligence; CA-125, cancer antigen 125; ctDNA, circulating tumor DNA; EPIRM-OC, Epigenetic Risk Markers in Ovarian Cancer study; MCED, multi-cancer early detection; miRNA, microRNA; MRD, minimal residual disease; N/A, not applicable; NCT, National Clinical Trial; pM, picomolar; STIC-2, Serial Testing to Identify Clonal Hematopoiesis study; STRIVE, study name for early cancer detection; TEP, tumor-educated platelet; TME, tumor microenvironment; VAF, variant allele frequency.ALDH1, aldehyde dehydrogenase 1; ATM, ataxia telangiectasia mutated; BCL2, B-cell lymphoma 2; BET, bromodomain and extra-terminal motif; BRCA, breast cancer gene; BRD4, bromodomain-containing protein 4; CAF, cancer-associated fibroblast; CDK, cyclin-dependent kinase; CLK, CDC-like kinase; ctDNA, circulating tumor DNA; CXCR2, C-X-C motif chemokine receptor 2; DNMT, DNA methyltransferase; DRP1, dynamin-related protein 1; ECM, extracellular matrix; EZH2, enhancer of zeste homolog 2; FASN, fatty acid synthase; FDG-PET, fluorodeoxyglucose positron emission tomography; GLS1, glutaminase 1; H3K27ac, histone H3 lysine 27 acetylation; H3K27me3, histone H3 lysine 27 trimethylation; H3K4me3, histone H3 lysine 4 trimethylation; HCQ, hydroxychloroquine; HDAC, histone deacetylase; HIF, hypoxia-inducible factor; IHC, immunohistochemistry; IL, interleukin; LC3B, microtubule-associated protein 1 light chain 3 beta; MDSC, myeloid-derived suppressor cell; MFN, mitofusin; mTOR, mechanistic target of rapamycin; NCT, National Clinical Trial; NOTCH, neurogenic locus notch homolog; OPA1, optic atrophy 1; OXPHOS, oxidative phosphorylation; PARPi, PARP inhibitor; PDH, pyruvate dehydrogenase; PGC1α, peroxisome proliferator-activated receptor gamma coactivator 1-alpha; RAD51, RAD51 recombinase; ROS, reactive oxygen species; SA-β-gal, senescence-associated beta-galactosidase; SASP, senescence-associated secretory phenotype; SF3B1, splicing factor 3B subunit 1; SRSF2, serine/arginine-rich splicing factor 2; TFEB, transcription factor EB; Treg, regulatory T cell; U2AF1, U2 small nuclear RNA auxiliary factor 1; ULK1, unc-51 like autophagy activating kinase 1; WNT, wingless/integrated; YAP/TAZ, Yes-associated protein/transcriptional coactivator with PDZ-binding motif.

Multi−analyte liquid biopsy strategies that integrate circulating tumor DNA (ctDNA) mutations, methylation, and proteins are rapidly progressing toward clinical use, with reviews in ovarian cancer highlighting their potential for early detection, minimal residual disease (MRD) tracking, and resistance profiling through serial sampling and dynamic monitoring (249, 260). Prospective data using mutation−targeted plasma assays show ctDNA can signal recurrence earlier than conventional markers like CA−125 and imaging, detecting relapse a median of 49 days before CA−125 rise and 7 days before radiologic confirmation in targeted droplet digital PCR studies, with all recurrent cases ctDNA−positive and no signals in recurrence−free patients (261). Complementary multi−analyte panels combining ctDNA with proteins (e.g., CA−125, HE4) can raise early detection sensitivity from 79–86% (single markers) to about 94% while maintaining high specificity, supporting a role beyond tissue biopsy in real−time surveillance (262). Together, these advances position liquid biopsy not just as an adjunct but as a potential primary tool for earlier recurrence detection, MRD monitoring, and resistance surveillance in ovarian cancer care (261, 263).

The scope of liquid biopsy extends beyond ctDNA. Circulating tumor cells (CTCs) can be isolated for functional assays and single-cell multi-omics, though technical isolation hurdles remain (264). Extracellular vesicles (e.g., exosomes) carry tumor-specific proteins, RNA, and DNA (264, 265), while circulating cell-free RNA and microRNAs offer complementary biomarkers for tracking disease dynamics (265). Integrating multiple analytes provides a more comprehensive tumor portrait.

Advanced technologies like droplet digital PCR, next-generation sequencing, and methylation analysis are enhancing sensitivity and specificity for applications from early diagnosis to managing refractory disease (264). These tools enable precise treatment response assessment, clonal evolution tracking, and MRD detection (265). Significant challenges persist, however, including the low abundance of tumor-derived material, interference from clonal hematopoiesis (especially in older patients), and the urgent need for standardized analytical and clinical validation to guide routine implementation (264, 265).

### 6.2 Functional drug sensitivity testing

Functional drug sensitivity testing using patient-derived ex vivo models bridges the gap between molecular profiling and clinical response by directly assessing the efficacy of therapeutic agents on a patient’s own tumor cells. Conditionally reprogrammed cell (CRC) culture enables the rapid propagation of primary tumor cells while preserving their inherent genetic and clonal diversity. This method has demonstrated high concordance between *in vitro* drug sensitivity results and clinical outcomes in cancers like colorectal carcinoma, and has been successfully applied in bladder cancer to identify effective treatments, including novel agents, offering a critical strategy for tumors lacking clear genomic drivers (266, 267).

Patient-derived organoids (PDOs) have emerged as a particularly powerful platform. These three-dimensional *in vitro* models faithfully recapitulate the histological architecture, molecular features, and cellular heterogeneity of the original tumor, with genomic concordance rates as high as 94% (268, 269). PDOs can be established from minimal tissue, such as core needle biopsies, and screened against comprehensive drug panels, yielding actionable therapeutic hits for over 90% of samples (269). The predictive clinical value is substantial, with chemograms derived from PDOs demonstrating 75% sensitivity and specificity for forecasting patient treatment response (269).

Recent technological advances are addressing key barriers to clinical implementation of patient-derived organoid (PDO) functional testing: microfluidic and organ-on-a-chip systems now enable high-throughput drug screening with minimal tissue and faster turnaround, while integrated sensors and automated imaging streamline continuous, *in situ* viability assessment and machine-learning–based response prediction; prospective studies show microfluidic platforms can deliver chemosensitivity readouts within roughly 1–2 weeks with strong concordance to clinical response, supporting feasibility for timely decision-making in oncology (270). Furthermore, sensor-integrated organ-on-chip platforms provide automated, continual monitoring that reduces hands-on time and enhances reproducibility for PDO assays at scale (271).

Importantly, recent prospective studies suggest clinical utility of patient-derived organoid (PDO)-guided therapy. In the phase II ORGANO trial for platinum-resistant ovarian cancer, PDO-guided treatment improved progression-free survival versus physician’s choice chemotherapy (median 5.2 vs. 3.8 months; HR 0.71; 95% CI 0.52–0.96; *P* = 0.03) (272). Similarly, in recurrent endometrial cancer, the PREDICT-END study reported higher objective response with PDO-guided therapy compared to empiric therapy (42% vs 28%) in a cohort of 87 patients, supporting the feasibility and translational relevance of PDOs for treatment selection (273).

However, significant challenges remain. Intratumoral heterogeneity is a major confounder, as PDOs derived from different regions of the same tumor can exhibit distinct molecular and drug-response profiles (274). The current turnaround time for establishing, expanding, and screening PDOs a median of 6 weeks poses a barrier for timely clinical decision-making (269). Despite these hurdles and the technical complexities of culture optimization, the global expansion of PDO biobanks underscores the immense potential of this approach to guide personalized therapy, especially for patients with rare or treatment-resistant cancers (268).

### 6.3 Innovative clinical trial designs

The molecular stratification of cancer has rendered traditional, histology-driven phase III trials inadequate due to their protracted timelines, rigid protocols, and inability to efficiently evaluate biomarker-defined subgroups (275). This has driven a necessary re-innovation in trial methodology, characterized by flexible, biomarker-integrated designs. These include: (1) basket trials, which test a single targeted therapy across multiple cancer types harboring a common molecular alteration; (2) umbrella trials, which evaluate multiple targeted therapies within a single cancer type, assigning patients to different arms based on their molecular profile; and (3) platform trials, which operate under a master protocol that can be modified to add, drop, or modify treatment arms in response to accumulating data (275, 276).

Platform trials represent a particularly transformative paradigm. Functioning as perpetual research infrastructures rather than fixed studies, they offer continuous learning and adaptation. The pioneering I-SPY 2 trial in breast cancer exemplifies this model, utilizing biomarker stratification, Bayesian response-adaptive randomization, and seamless phase II/III design to efficiently graduate multiple novel agents for confirmatory testing (277, 278). Its adaptive framework allows for the early termination of ineffective arms and the prompt integration of new therapies based on emerging biomarker data (277). This approach demonstrates superior efficiency, typically requiring fewer patients and achieving conclusions faster than traditional trials, with candidates showing a higher predicted likelihood of phase III success (278).

The RAINBO (Refining Adjuvant treatment IN endometrial cancer Based On molecular profile) program is a platform of four parallel phase III trials assigning adjuvant therapy by TCGA molecular class. RAINBO-Green enrolls patients with *POLE*-ultramutated tumors and tests de-escalation to observation alone, leveraging the excellent prognosis of this subgroup to safely avoid overtreatment. RAINBO-Blue focuses on dMMR/MSI-H tumors, evaluating adjuvant durvalumab versus placebo to capitalize on the immunogenicity of this subgroup. RAINBO-Red targets p53-abnormal/copy-number high tumors, testing chemotherapy plus olaparib versus chemotherapy alone to exploit DNA repair vulnerabilities in this high-risk population. RAINBO-Amber addresses the heterogeneous NSMP subgroup, comparing adjuvant chemotherapy versus observation to refine management in this intermediate-risk group (279, 280).

This molecularly driven strategy reflects the integration of TCGA subgroups into contemporary management, with emerging evidence that PD-(L)1 blockade benefits dMMR disease and that PARP inhibition may add value in selected contexts, supporting the rationale of these tailored adjuvant approaches (279, 280). The RAINBO program employs a master protocol to concurrently test four biomarker-driven hypotheses, aligning adjuvant therapy with molecular classes p53-abnormal and MMRd for escalation, and *POLE*-mutated for de-escalation thereby translating genomic taxonomy into clinical decisions while leveraging shared infrastructure for efficiency and global standardization (279). Early implementation studies and cooperative group data show strong feasibility and adoption of molecular profiling in routine care, with >90% of clinicians rating p53, MMR, and *POLE* as important for management despite variable access, supporting real-world translational uptake consistent with RAINBO’s framework (33).

However, implementation presents challenges, including complex statistical and operational logistics, the need for sophisticated central infrastructure, and regulatory adaptation to novel endpoints. The early termination of pexidartinib in I-SPY 2 due to hepatic toxicity also highlights the critical importance of rigorous safety monitoring within these adaptive frameworks (281). Despite these hurdles, platform trials and related innovative designs are crucial for maximizing information extraction per patient, accelerating the validation of precision therapies, and effectively matching the right drug to the right biomarker-defined patient population.

### 6.4 Artificial intelligence in precision oncology: from data integration to clinical decision support

The practice of precision oncology generates immense, multi-dimensional datasets from multi-omics and digital pathology to medical imaging and electronic health record that surpass the integrative capacity of traditional analysis. This complexity presents a critical opportunity for artificial intelligence (AI) and machine learning to transform data into clinical insight (282).

AI excels at discerning non-linear patterns and subtle correlations across these disparate data modalities. Key applications include predicting treatment response, refining prognostic stratification, elucidating resistance mechanisms, and enhancing early diagnosis through advanced radiomics and deep learning models (282). A core strength lies in its ability to process and integrate large-scale genomic and transcriptomic data, facilitating more nuanced and data-driven therapeutic decisions as biological information becomes increasingly digitized (282, 283).

Recent AI advances in gynecologic oncology pathology show strong potential for molecular classification directly from routine H&E slides. In endometrial cancer, interpretable whole-slide pipelines have reached class-wise AUROC of 0.928 for p53-abnormal, 0.844 for MMR-deficient, and 0.849 for POLE-mutated tumors, suggesting near-molecular performance where sequencing is unavailable (284). Complementary work reports AUROC ≈0.91 for MMR status using ResNet50 on tile-level images, reinforcing feasibility for prescreening in resource-limited settings (285). For ovarian cancer histotyping, vision-transformer and CNN frameworks demonstrate robust discrimination of high-grade serous versus other subtypes, with reported accuracies in the high-80% range, supporting deployment where subspecialist review is scarce (286, 287).

In prognostic modeling, multimodal AI approaches integrating histopathology, genomics, and clinical data have begun to outperform traditional nomograms. Recent endometrial cancer studies show that combining H&E whole-slide image features with clinical variables improves survival discrimination, with reported concordance indices (C-index) of 0.82–0.88 versus ~0.73–0.80 for clinical staging alone, identifying ostensibly early-stage patients with poor outcomes and suggesting occult high-risk features missed by conventional assessment (288, 289).

Radiomics-based models show promise for treatment response prediction. In locally advanced cervical cancer, hybrid MRI radiomics integrating handcrafted and deep features with clinical data predicted pathologic complete response with an AUC of 0.80 (75% sensitivity, 66% specificity) (290). Dynamic contrast-enhanced MRI parameters stratified early responders, with multi-parameter combinations achieving AUC 0.95 (87% sensitivity, 96% specificity) (291). Diffusion and perfusion biomarkers (apparent diffusion coefficient, Ktrans/Kep) combined yielded AUCs of 0.90 in early-assessment settings (292, 293).

Emerging applications include natural language processing (NLP) for automated extraction of biomarker status from electronic health records, with recent studies demonstrating >95% accuracy in identifying hormone receptor (ER/PR), MMR, and HER2 status from unstructured pathology reports, enabling scalable real-world evidence generation and more efficient clinical trial screening (294, 295).

Despite notable progress, several challenges temper enthusiasm. Cross-site generalizability remains limited; multicenter evaluations show meaningful performance drops from internal to external testing due to domain shift, underscoring the need for diverse training and rigorous out-of-distribution validation (296, 297). The “black box” problem persists, slowing clinical adoption; even when internal AUCs exceed 0.98, external AUCs can drop to ~0.95, emphasizing the need for transparent modeling, calibration checks, and independent validation before deployment (298, 299). Building local validation infrastructure is essential to assess accuracy, calibration, and equity prior to workflow integration (300, 301). Federated or multi-center collaborations aim to bolster robustness without centralizing data, yet significant external performance gaps can persist, reinforcing that prospective, independent validation remains a prerequisite for regulatory-grade, primary diagnostic use (296, 297).

Looking forward, the integration of AI-based clinical decision support tools into routine practice will require not only technical validation but also careful attention to workflow integration, clinician trust, and potential algorithmic bias. The ongoing AI-ENDO trial (NCT05879627) is one of the first randomized controlled trials evaluating whether an AI-based treatment recommendation system improves outcomes in recurrent endometrial cancer; results expected in 2026 will provide critical evidence for the clinical utility of these approaches (302). Parallel reviews in gynecologic oncology emphasize human-in-the-loop design, explainability, prospective trials, and equity-centered deployment as prerequisites for trust and safe adoption (303, 304).

## 7 Conclusion

Precision oncology has fundamentally transformed gynecologic cancer care, shifting the paradigm from histology-based to molecularly-driven diagnosis and treatment. The success of biomarker-guided therapies, such as PARP inhibitors for HRD-positive ovarian cancer and immune checkpoint blockers for dMMR/MSI-H endometrial cancer, validates targeting specific molecular vulnerabilities. However, the persistent challenge of therapeutic resistance necessitates a deeper understanding of tumor evolution and the integration of dynamic biomarkers like circulating tumor DNA for adaptive treatment strategies. Crucially, ensuring equitable global access to these advanced diagnostics and therapies is imperative to prevent widening health disparities, particularly as temporal trends reveal a growing burden in low- and middle-income countries.

The future of the field will be shaped by the convergence of artificial intelligence for multi-omics integration, novel therapeutic modalities like next-generation ADCs and cellular therapies, and innovative adaptive trial designs such as the RAINBO program. Furthermore, advancing early detection through liquid biopsy could shift precision interventions to earlier, more curable disease stages. Ultimately, overcoming implementation barriers related to cost, access, and education is essential to democratize these advances. By evolving toward a model of continuous molecular monitoring and adaptive therapy, precision oncology aims to transform advanced gynecologic cancers into chronically manageable conditions, moving closer to the goal of truly personalized and accessible care for every patient.
